# Supplementary material for: Augmenting interictal mapping with neurovascular coupling biomarkers by structured factorization of epileptic EEG and fMRI data
Source: Neuroimage. 2021 Mar;228:117652. doi: 10.1016/j.neuroimage.2020.117652 (PMC7903163; doi:10.1016/j.neuroimage.2020.117652)
Supplement: Supplementary Data S1 — Supplementary Raw Research Data. This is open data under the CC BY license http://creativecommons.org/licenses/by/4.0/ [file mmc1.pdf]

Augmenting interictal mapping with neurovascular coupling biomarkers by structured factorization of epileptic EEG and fMRI data

Simon Van Eyndhoven[a,*], Patrick Dupont[b,c], Simon Tousseyn[d], Nico Vervliet[a], Wim Van Paesschen[e,f], Sabine Van Huffel[a], Borbála Hunyadi[g]

[a]KU Leuven, Department of Electrical Engineering (ESAT), STADIUS Center for Dynamical Systems, Signal Processing and Data Analytics
[b]Laboratory for Cognitive Neurology, Department of Neurosciences, KU Leuven, Leuven, Belgium
[c]Leuven Brain Institute, Leuven, Belgium
[d]Academic Center for Epileptology, Kempenhaeghe and Maastricht UMC+, Heeze, The Netherlands
[e]Laboratory for Epilepsy Research, KU Leuven, Leuven, Belgium
[f]Department of Neurology, University Hospitals Leuven, Leuven, Belgium
[g]Circuits and Systems Group (CAS), Department of Microelectronics, Delft University of Technology, Delft, the Netherlands

Supplement: sCMTF signatures for all patients

We show the EEG spatial, temporal and spectral signatures, HRF waveforms, and fMRI spatial maps of significant IED (de)activation and HRF variability for all patients (except patients 3 and 10, whose results have been analyzed in the main text).

Patient 1

We analyze the solution with $\hat{R} = 6$ sources. Figure 1 shows the EEG signatures and HRF waveforms. One of the sources is highly correlated to the MWF reference (in green), which was already known from Table B.3. This IED-related source had a typical low-frequency spectrum, which is expected for the typical spike-and-wave interictal discharges. The topography is relatively diffuse, although the highest amplitudes are mostly in the left hemisphere. This is in accordance with the lateralization of ictal onset zone (left temporal lobe, cfr. Table 1). There are some noteworthy observations to be made about some of the other components. The fourth has an unusually sharp spectrum, is mainly localized on two nonadjacent center electrodes, and is sustained for a single period of many seconds Hence, this component likely captured an artifact (of yet unknown origin), although we spotted no large-amplitude changes in the EEG itself. Similarly, the third source is only present at one frontal electrode, and exists in a frequency range above 20 Hz. It might represent a muscle artifact, e.g., due to frowning or twitching of some muscles in the forehead. The HRFs of all ROIs are shown in Figure 1b. Two of the basis functions seem to have converged to a very similar waveform, which is an unfortunate possibility if two initial HRFs are too close to the same local optimum in their respective parameters. This reduces the expressive power of the basis set, which is clearly visible, since many ROIs have a nearly identical HRF. One of the twenty ROIs with the highest-entropy HRF overlapped the IOZ, although clearly this HRF (bold line) is not among the most dissimilar waveforms for this patient. This is also visible in Figure 2: both the HRF entropy and extremity maps show a small overlap with the delineated IOZ. Despite the good correspondence in the EEG domain, no significant (de)activation of the IED-component is found inside the IOZ.

Patient 2

We analyze the solution with $\hat{R} = 3$ sources, and show the results in Figure 3 and 4. As for patient 1, we found a source which is strongly correlated to the MWF envelope, and which had a mostly low-frequency behavior characteristic for spikes. The topography is mostly uninformative, and does not clearly correspond to the patient's clinical data. The third source is mostly present at both sides of the head, is very sparsely active in time, and has a high-frequency content: this is most likely an artifact due to the neck muscles. Again, there is one of the highest-entropy HRFs which belongs to a ROI in the IOZ. Now, the waveform is clearly resolved from the other HRFs, through the strong initial dip (before 0 seconds). Such a dip is sometimes observed in HRFs, but its underlying physiological mechanism is not yet fully understood. It is possible that this dip reflects altered vascular autoregulation near the IOZ (cfr. the explanation in the Section 1 of the main text), or a rapid depletion in oxygen due to IED generation (before the IED becomes visible on the EEG). Figure 4 furthermore shows that the IED-related component is significantly active in parts of the IOZ, and deactive in others. As mentioned earlier, this deactivation may or may not be due to errors in sign correction. Interestingly, the ROI with the high alteration in neurovascular coupling is distinct from both the activated and deactivated ROIs.

*Corresponding author
Email address: simon.vaneyndhoven@kuleuven.be, simon.vaneyndhoven@gmail.com

Patient 4

We analyzed the solution with $\hat{R} = 4$ sources, and show the results in Figure 5 and 6. There is one source which is mostly correlated to the reference (but not extremely, see also Table B.3). This source had a right-temporal focus, conform the diagnosis in Table 1. The second source illustrates the phenomenon of an erroneous sign exchange between the spatial and spectral profiles. Also one of the HRFs has a negative polarity, which is a failure of the sign correction procedure (in this case, because there is exceptionally no positive overshoot). However, the HRF variability metrics are still interpretable, and indeed two ROIs among the ones with the highest-entropy HRFs overlap with the IOZ. The IED component is significantly active in a tiny portion of the IOZ (cfr. Figure 6). The second source is significantly active in symmetrical parts of the parietal lobe. Given its ongoing fluctuation over time, we hypothesize that this source captures a resting state network (RSN).

Patient 5

We analyze the solution with $\hat{R} = 5$ sources, and show the results in Figure 7 and 8. There is a clear IED-related component, with a very high correlation to the MWF reference, a typical spectrum, and an anterior-temporal focus, which corresponds very well to the patient's diagnosis (cfr. Table 1). The fifth source seems present at only one channel, and has spectral harmonic at ± 17 Hz and ± 34 Hz. One of these peaks is reminiscent of the fourth component in patient 1. As Figure 8 shows, the HRF entropy and extremity prove to be relatively precise indicators for the IOZ in this case, and also the significant IED activation and deactivation allow correct localization. In Figure 7, it is clear that some HRFs may still have the wrong sign, which means that the interpretation of 'active' and 'deactivated' is flipped in those ROIs. Hence, regions of significant deactivation are in fact significantly activated. The fourth source had a significant overlap with the auditory RSN, and its spectrum reveals activity in the β band.

Patient 6

We analyze the solution with $\hat{R} = 2$ sources, and show the results in Figure 9 and 10. One source is strongly correlated to the MWF, while the other source is likely an artifact, given its very sparse temporal profile. Both sources coincide at one high-amplitude peak, by which we infer that this is probably an artifactual period in the signal. Indeed, when inspecting the original EEG signals, we found high-frequency muscle artifacts at these times. This source also had no significant activation in its spatial map, which corroborates its non-neuronal origin. The IED-related source had a broader spectrum than most other cases, and an uninformative topography. None of the ROIs with high-entropy HRFs is located in the IOZ. The pseudo t-map provides correct localization of the IOZ, however.

Patient 7

We analyze the solution with $\hat{R} = 4$ sources, and show the results in Figure 11 and 12. We found a clear IED-related component, with a characteristic spectrum and a topography which is backed up by the patient's diagnosis (left anterior-temporal IOZ). The fourth source has a very similar topography and spectrum to the fifth source in patient 5. One HRF inside the IOZ had a high-entropy, and is distinguishable from the others by its very sluggish waveform, i.e., it is smeared out in time, with no sharp over- or undershoot. Also the pseudo t-map provided an accurate localization of the IOZ. Notably, in this patient, the extremity metric misses the deviating HRF in the IOZ (while the entropy metric picks it up). The second source overlapped with the frontal part of the default mode network (DMN), and is active in the α and low β bands.

Patient 8

We analyze the solution with $\hat{R} = 2$ sources, and show the results in Figure 13 and 14. We found two components which had correlated time courses. At the time of the peaks, we found higher-amplitude events in the EEG with dubious origin, hence they may or may not be artifacts. One of both components is more strongly correlated to the MWF, and its activation is concordant with the IOZ. The second component shows high overlap with the sensorimotor network. For this patient, none of the IOZ's ROIs had extreme values of either HRF metric.

Patient 9

We analyze the solution with $\hat{R} = 2$ sources, and show the results in Figure 15 and 16. In this patient, there is only a moderate correlation of a component with the MWF reference time course. This component's topography (left occipital) agrees with the clinical description, however. The HRF extremity (and not the entropy) is high in a small part of the IOZ. Both the significant IED activation and deactivation allow correct localization as well. The second source seemingly captured high-frequency oscillatory activity in the sensorimotor network, similar to the previous patient.

Patient 11

We analyze the solution with $\hat{R} = 2$ sources, and show the results in Figure 17 and 18. The IED-related source had a high correlation with the MWF reference, but an odd bimodal spectrum. Its EEG topography is very consistent with the clinical description. Both HRF extremity and entropy are useful indicators for the IOZ. The IED activation and deactivation maps each had a very small overlap with the IOZ. The second source is temporally sparse and captures high-frequency EEG variations, which we identified as muscle artifacts.

Patient 12

We analyze the solution with $\hat{R} = 2$ sources, and show the results in Figure 19 and 20. Again we observe an IED-related source and a seemingly artifactual source with a spectral peak near 34 Hz. Many of the high-entropy HRFs are highly noncausal, and are associated to ROIs inside the IOZ. Hence, with both HRF metrics, the highest-scoring ROIs provides good localization of the HRF. While there are no significantly active ROIs in the IOZ, there are several significantly deactivated ROIs, which may indicate that the sign standardization was not done flawlessly (cfr. also some of the negative-peaking HRFs for patient 10). Surprisingly, the second source had one significantly active ROI, which overlaps with the IOZ, but which did not match its EEG topography. Hence, the nature of this source remains ambiguous.

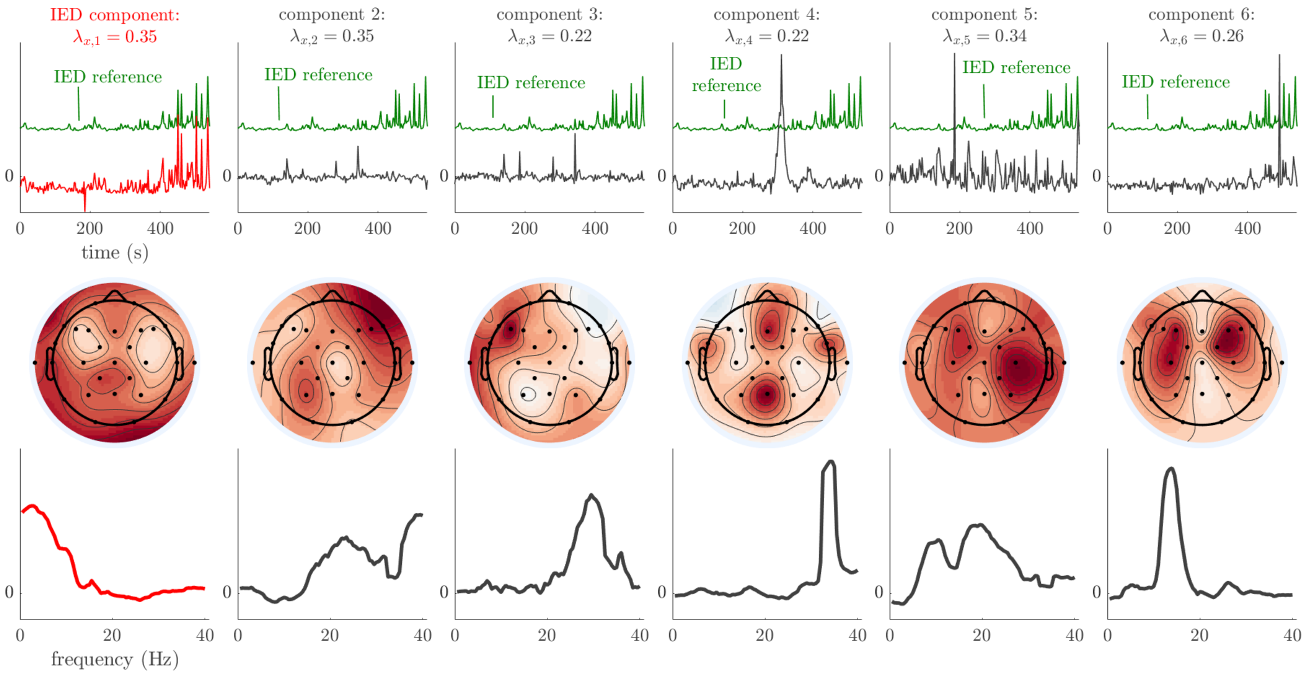

(a) Temporal ($\mathbf{s}_r$, top), spatial ($\mathbf{m}_r$, middle), and spectral ($\mathbf{g}_r$, bottom) profiles of the 6 sources in the EEG domain, and reference IED time course ($\mathbf{s}_{\text{ref}}$, in green).

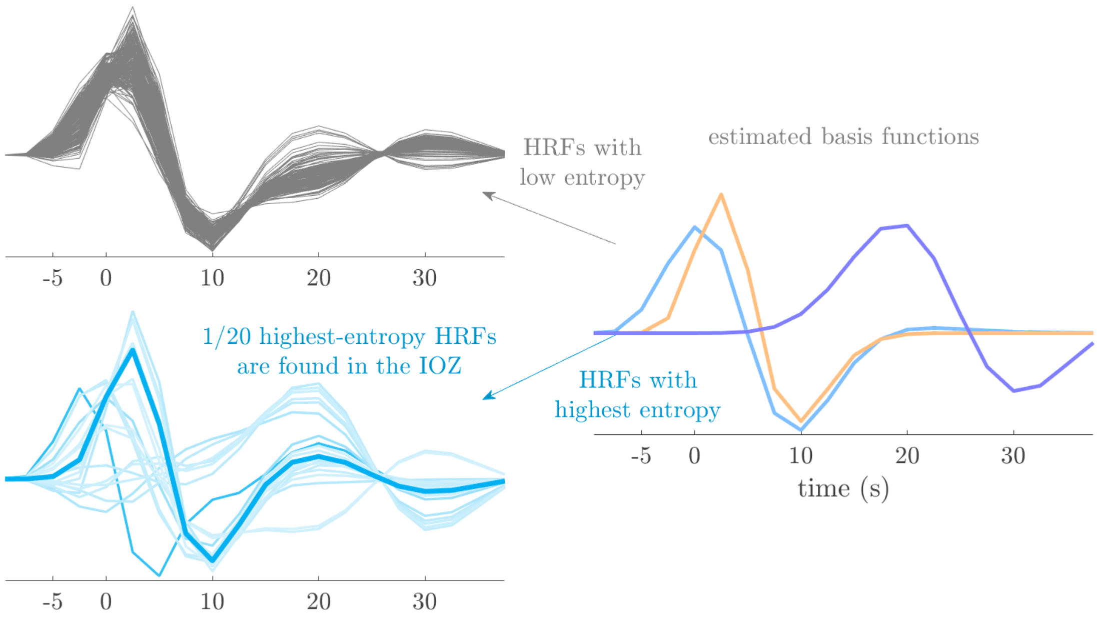

(b) Estimated HRFs with low and high entropy

Figure 1 Patient 1's estimated sources and neurovascular coupling parameters. **(a)** The IED-related component correlates well with the reference time course, and is mostly a low-frequency phenomenon. The fourth source is most likely an artifact (of unknown origin), picked up mostly by two central channels. **(b)** One of the ROIs with the highest-entropy HRFs belongs to the ictal onset zone (bold line, $p = 0.34$).

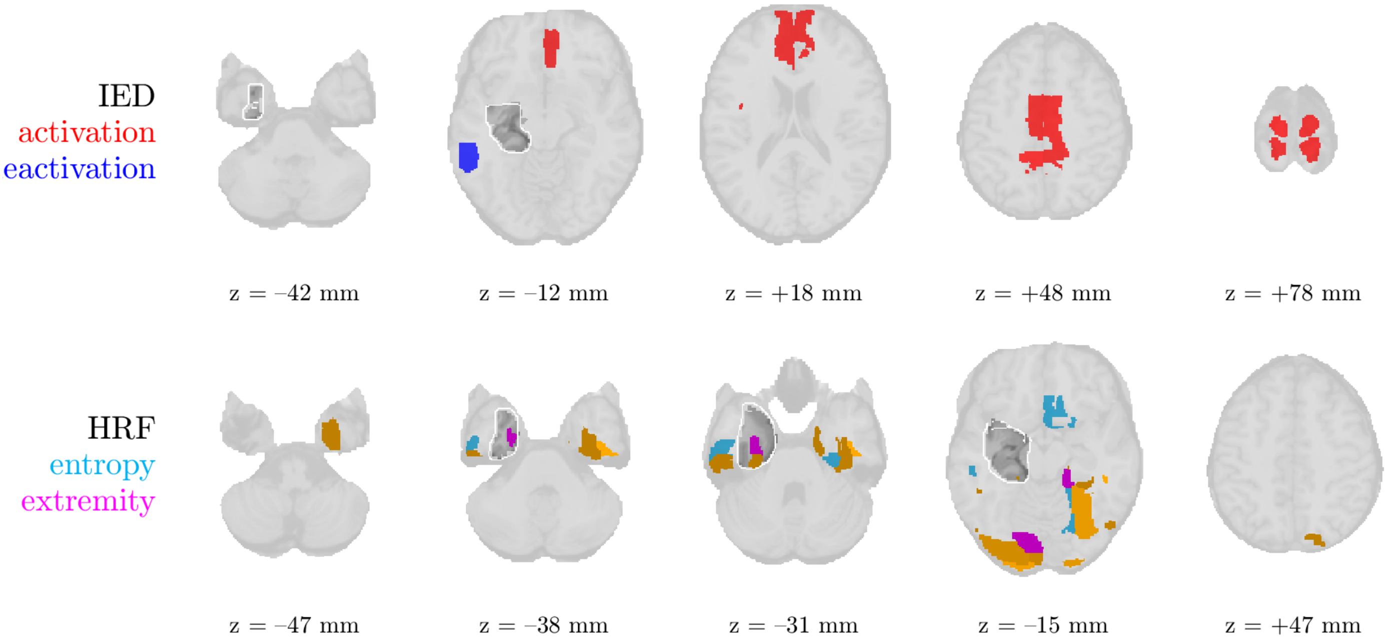

Figure 2 Patient 1's statistical nonparametric maps and HRF entropy/extremity maps. The ground truth ictal onset zone is highlighted in dark gray with a white contour. Regions with both high HRF entropy and high HRF extremity are colored in orange.

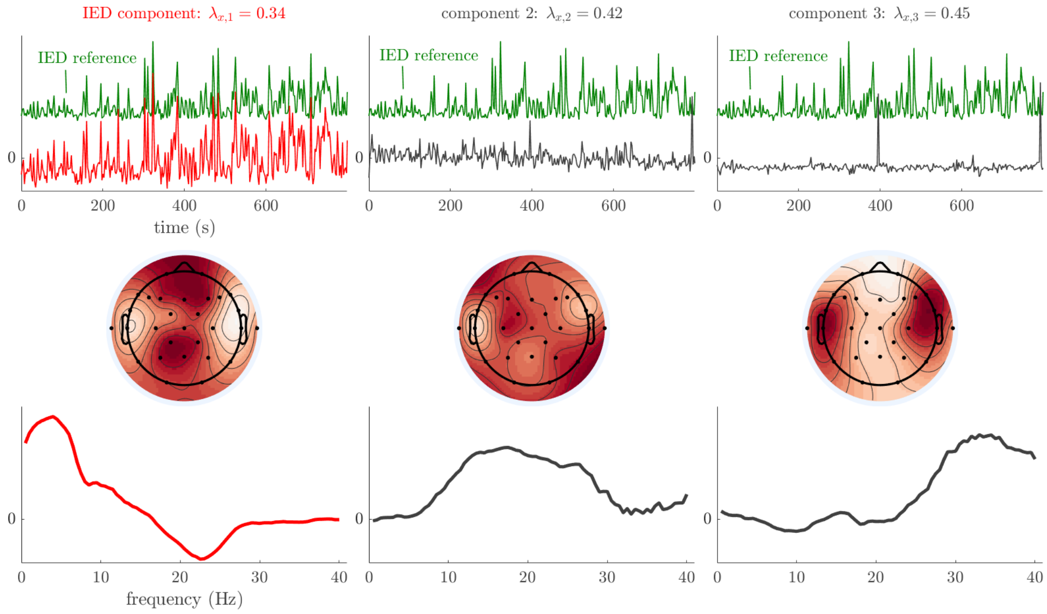

(a) Temporal ($\mathbf{s}_r$, top), spatial ($\mathbf{m}_r$, middle), and spectral ($\mathbf{g}_r$, bottom) profiles of the 3 sources in the EEG domain, and reference IED time course ($\mathbf{s}_{\text{ref}}$, in green).

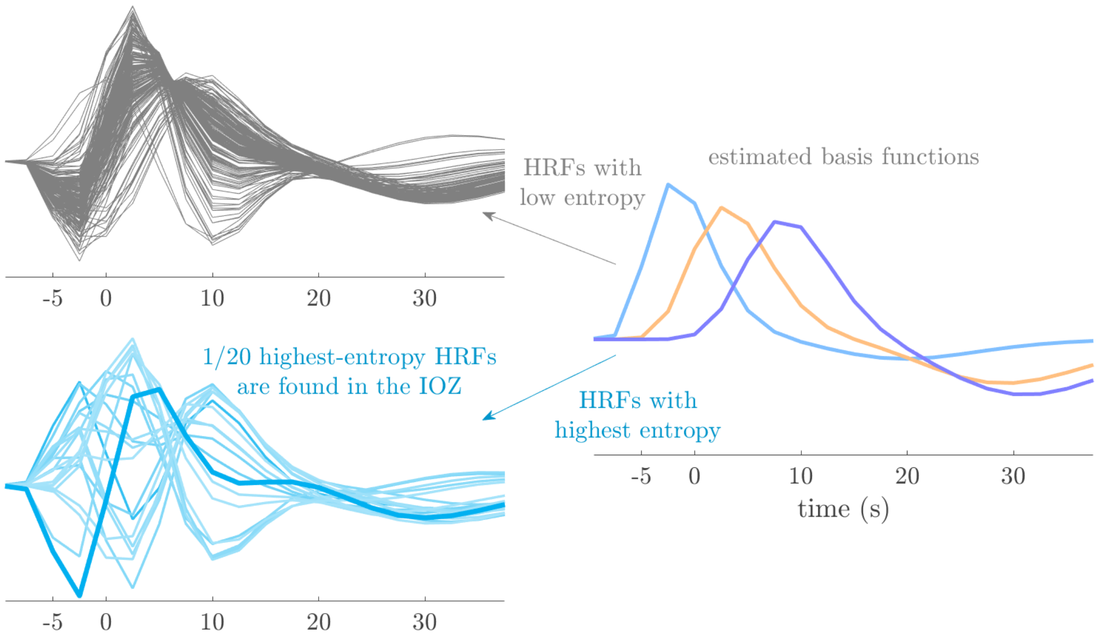

(b) Estimated HRFs with low and high entropy

Figure 3 Patient 2's estimated sources and neurovascular coupling parameters. **(a)** The IED-related component correlates well with the reference time course, and is mostly a low-frequency phenomenon. **(b)** One of the ROIs with the highest-entropy HRFs belongs to the ictal onset zone (bold line, $p = 0.59$).

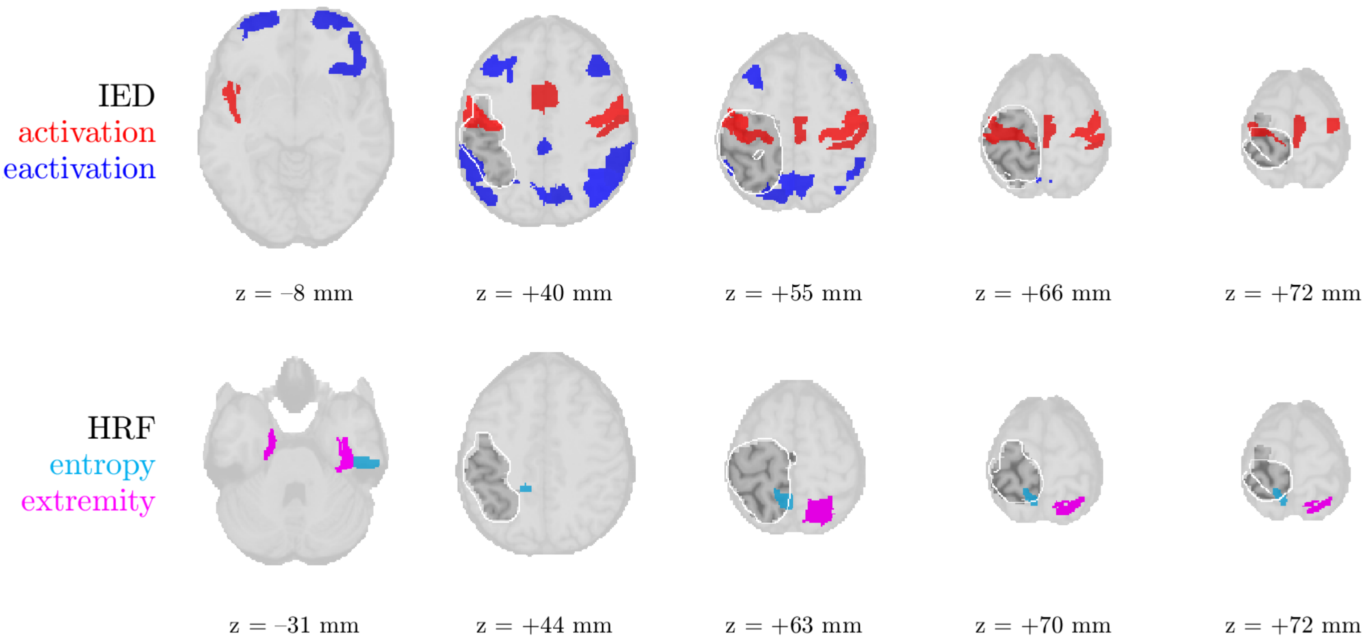

Figure 4 Patient 2's statistical nonparametric maps and HRF entropy/extremity maps. The ground truth ictal onset zone is highlighted in dark gray with a white contour. Regions with both high HRF entropy and high HRF extremity are colored in orange.

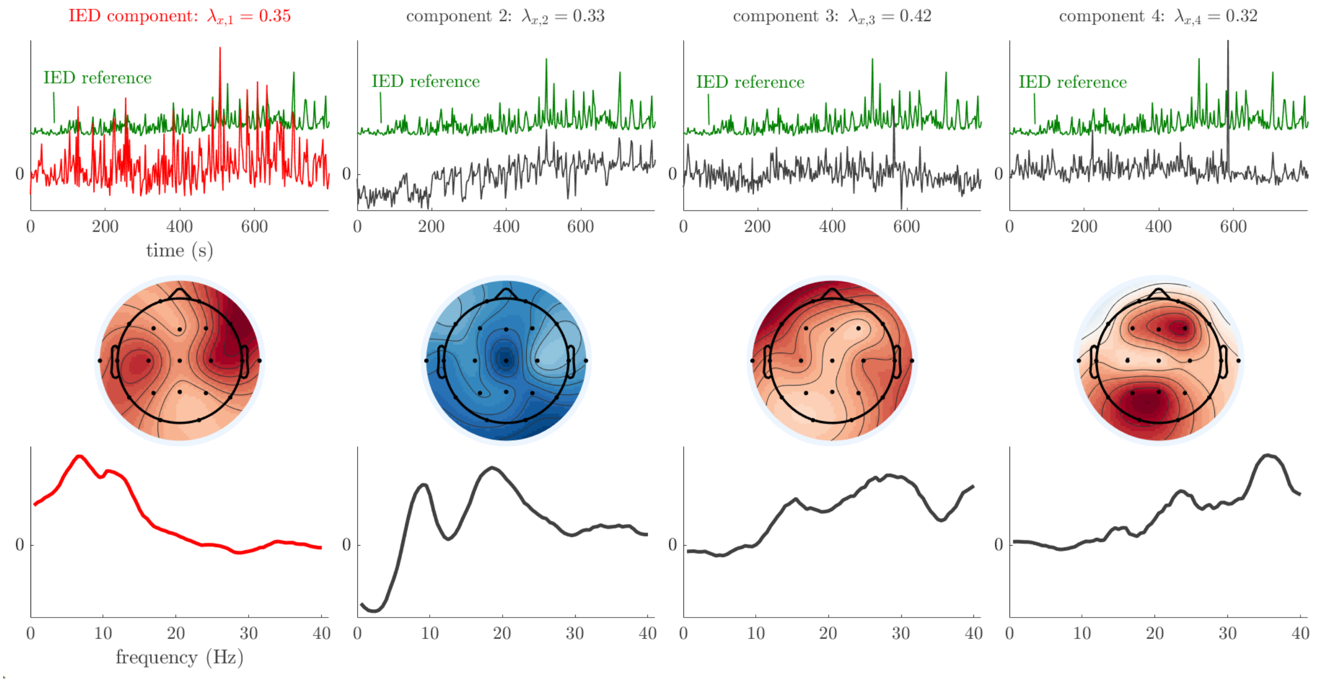

(a) Temporal ($\mathbf{s}_r$, top), spatial ($\mathbf{m}_r$, middle), and spectral ($\mathbf{g}_r$, bottom) profiles of the 4 sources in the EEG domain, and reference IED time course ($\mathbf{s}_{\text{ref}}$, in green).

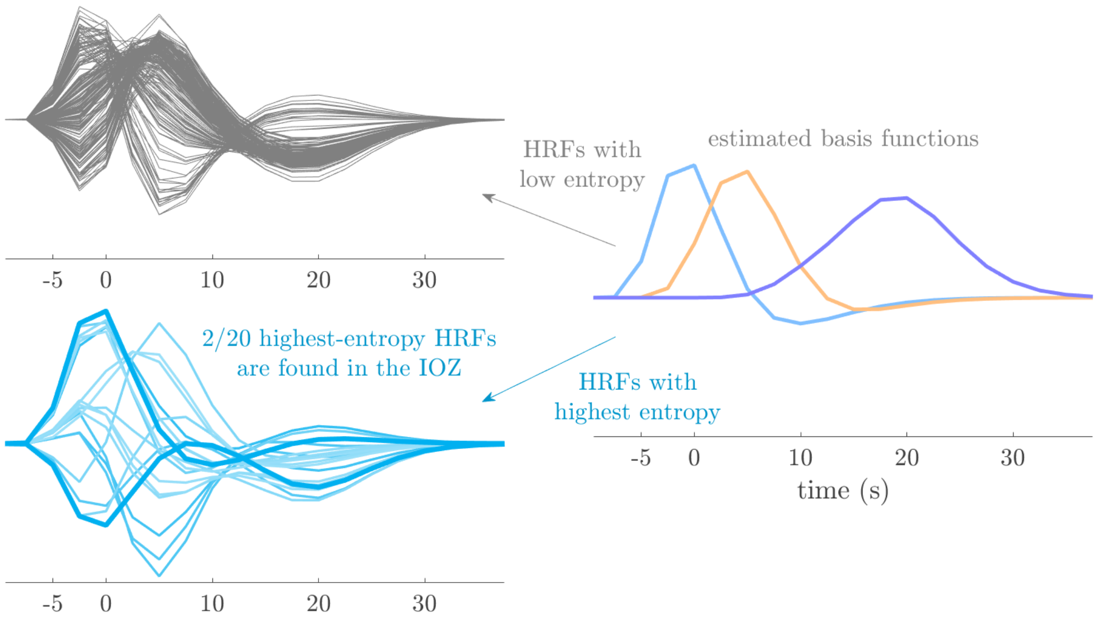

(b) Estimated HRFs with low and high entropy

Figure 5 Patient 4's estimated sources and neurovascular coupling parameters. **(b)** Two of the ROIs with the highest-entropy HRFs belong to the ictal onset zone (bold line, $p = 0.32$).

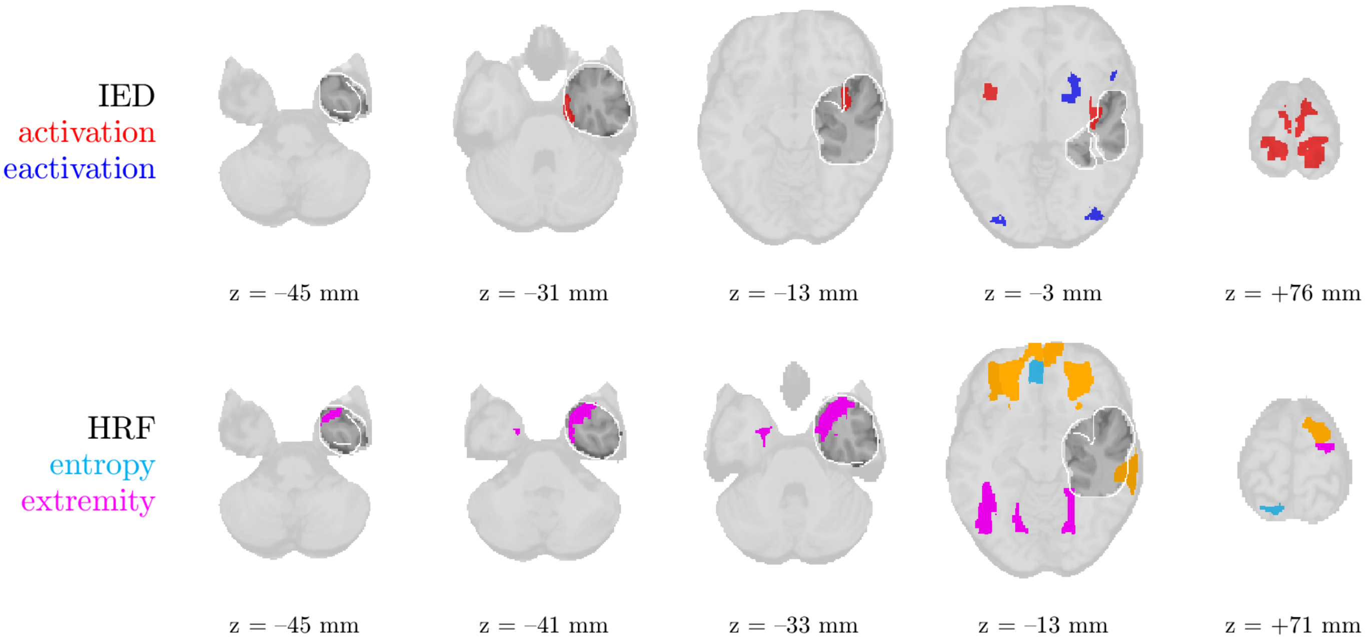

Figure 6 Patient 4's statistical nonparametric maps and HRF entropy/extremity maps. The ground truth ictal onset zone is highlighted in dark gray with a white contour. Regions with both high HRF entropy and high HRF extremity are colored in orange.

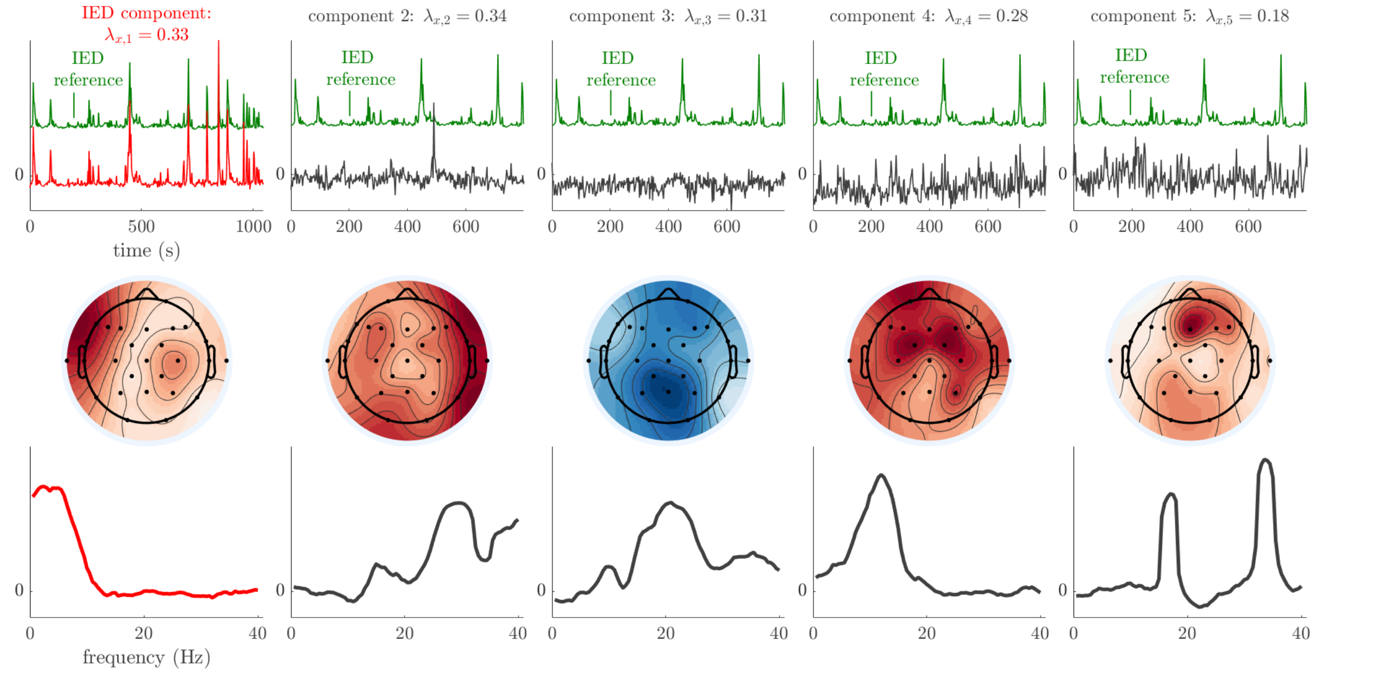

(a) Temporal ($\mathbf{s}_r$, top), spatial ($\mathbf{m}_r$, middle), and spectral ($\mathbf{g}_r$, bottom) profiles of the 5 sources in the EEG domain, and reference IED time course ($\mathbf{s}_{\text{ref}}$, in green).

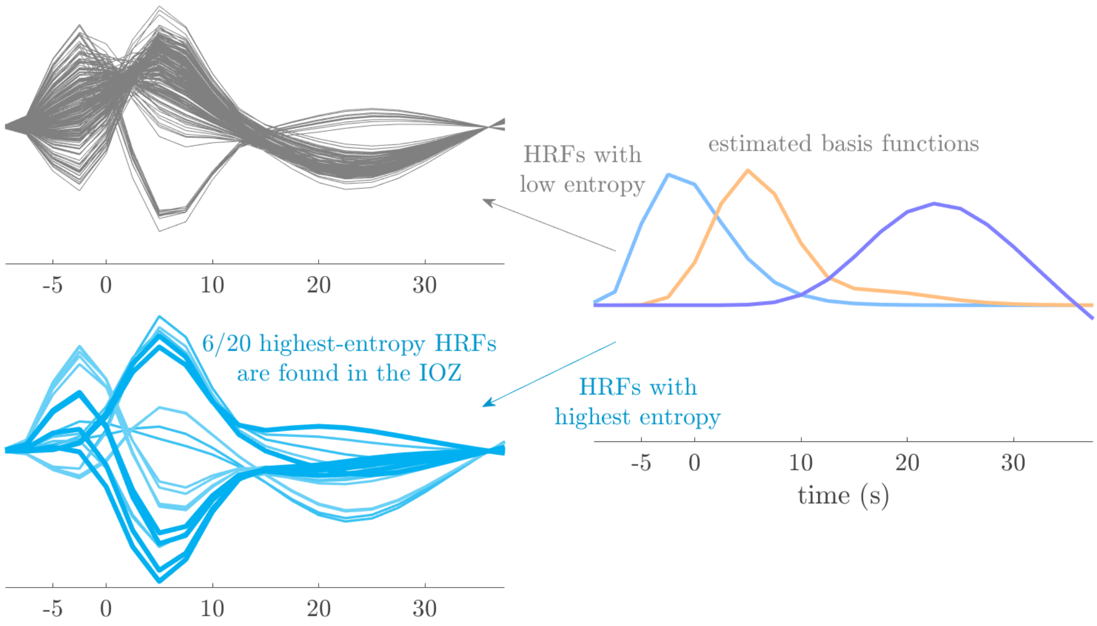

(b) Estimated HRFs with low and high entropy

Figure 7 Patient 5's estimated sources and neurovascular coupling parameters. **(b)** Six of the ROIs with the highest-entropy HRFs belong to the ictal onset zone (bold line, $p < 10^{-3}$).

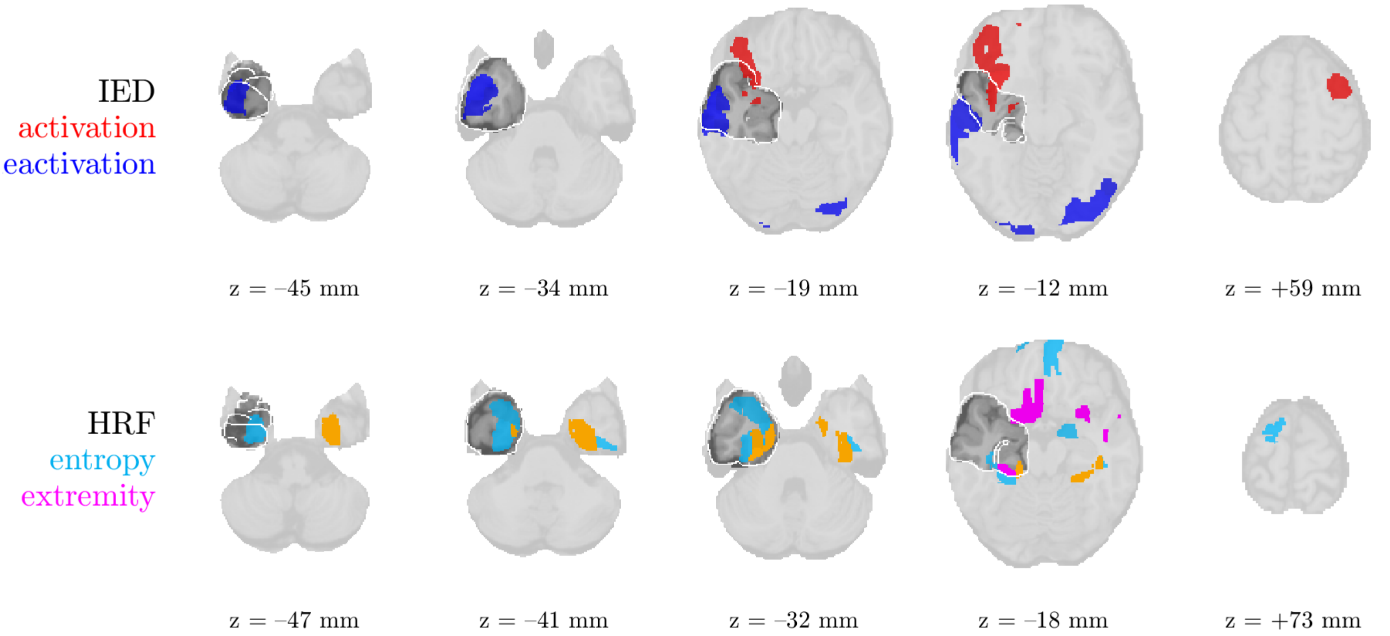

Figure 8 Patient 5's statistical nonparametric maps and HRF entropy/extremity maps. The ground truth ictal onset zone is highlighted in dark gray with a white contour. Regions with both high HRF entropy and high HRF extremity are colored in orange.

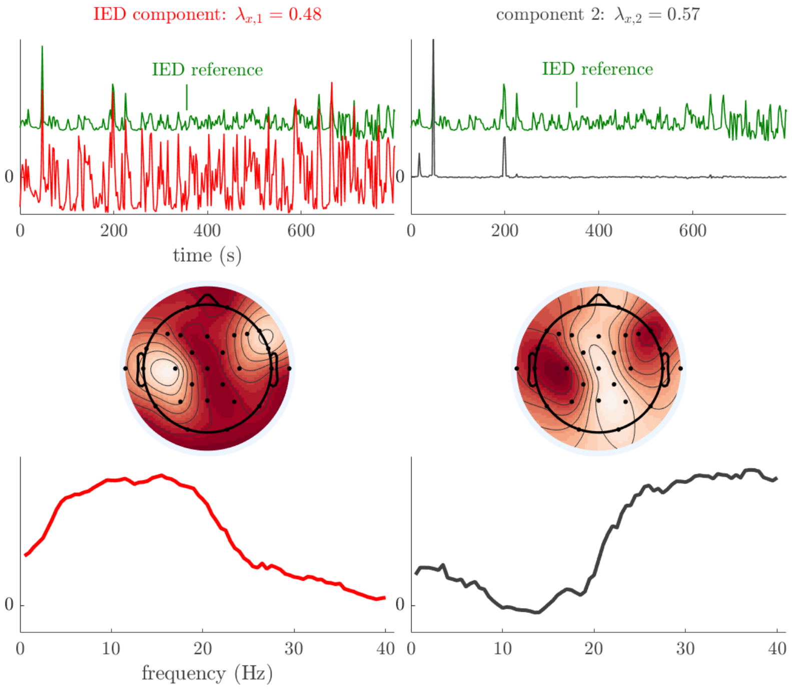

(a) Temporal ($\mathbf{s}_r$, top), spatial ($\mathbf{m}_r$, middle), and spectral ($\mathbf{g}_r$, bottom) profiles of the 2 sources in the EEG domain, and reference IED time course ($\mathbf{s}_{\text{ref}}$, in green).

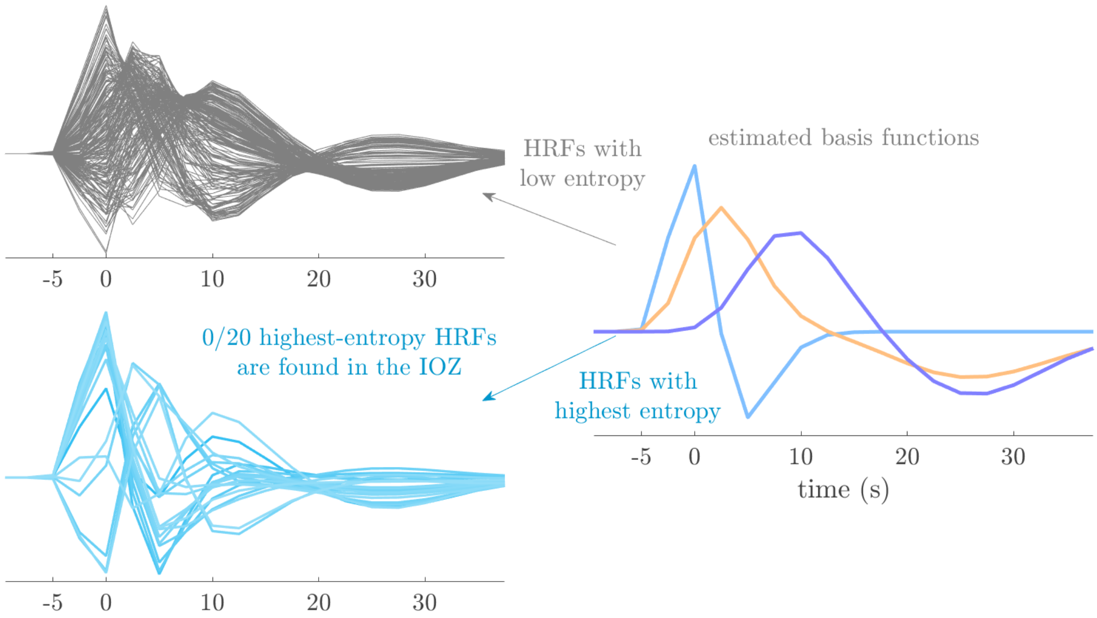

(b) Estimated HRFs with low and high entropy

Figure 9 Patient 6's estimated sources and neurovascular coupling parameters. **(b)** None of the ROIs with the highest-entropy HRFs belong to the ictal onset zone.

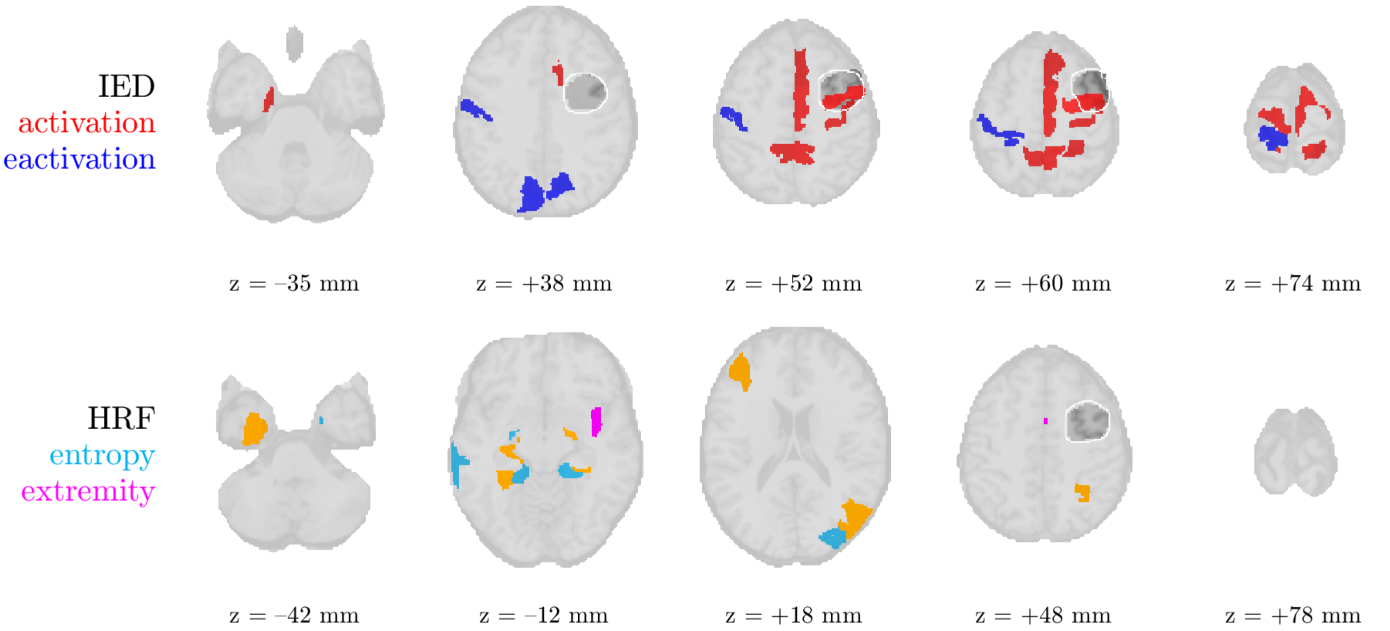

Figure 10 Patient 6's statistical nonparametric maps and HRF entropy/extremity maps. The ground truth ictal onset zone is highlighted in dark gray with a white contour. Regions with both high HRF entropy and high HRF extremity are colored in orange.

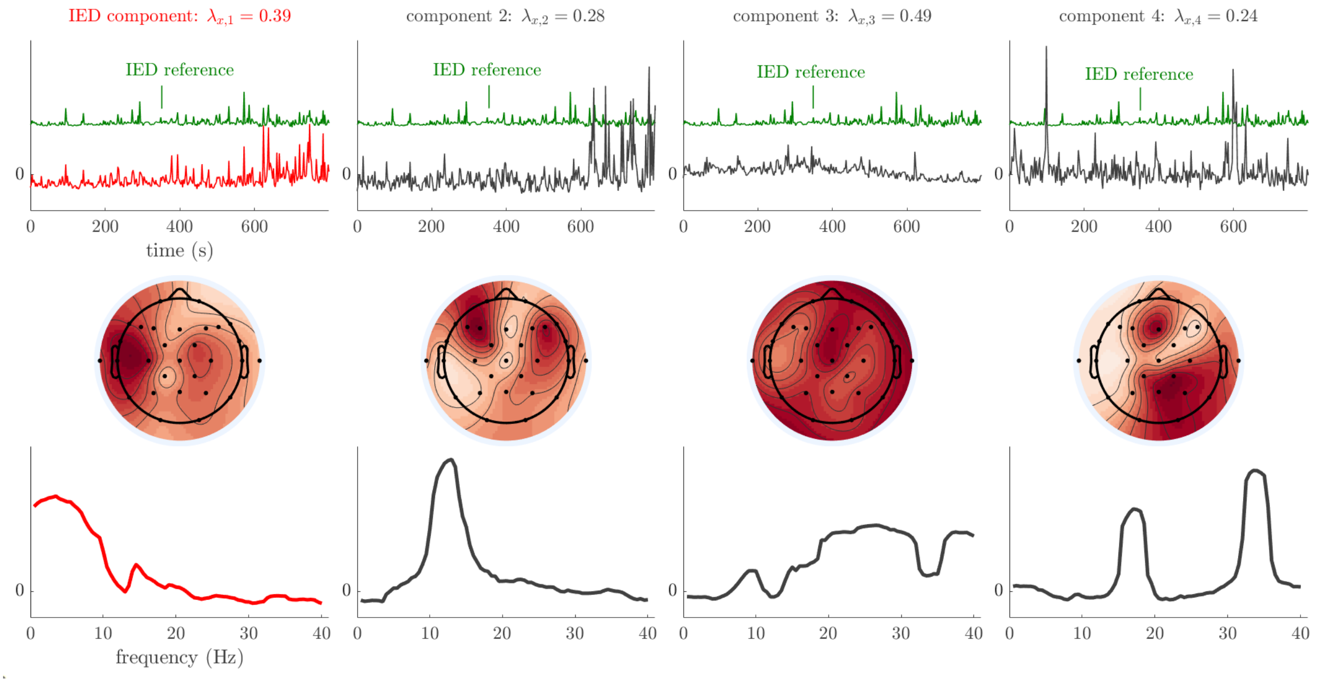

(a) Temporal ($\mathbf{s}_r$, top), spatial ($\mathbf{m}_r$, middle), and spectral ($\mathbf{g}_r$, bottom) profiles of the 4 sources in the EEG domain, and reference IED time course ($\mathbf{s}_{\text{ref}}$, in green).

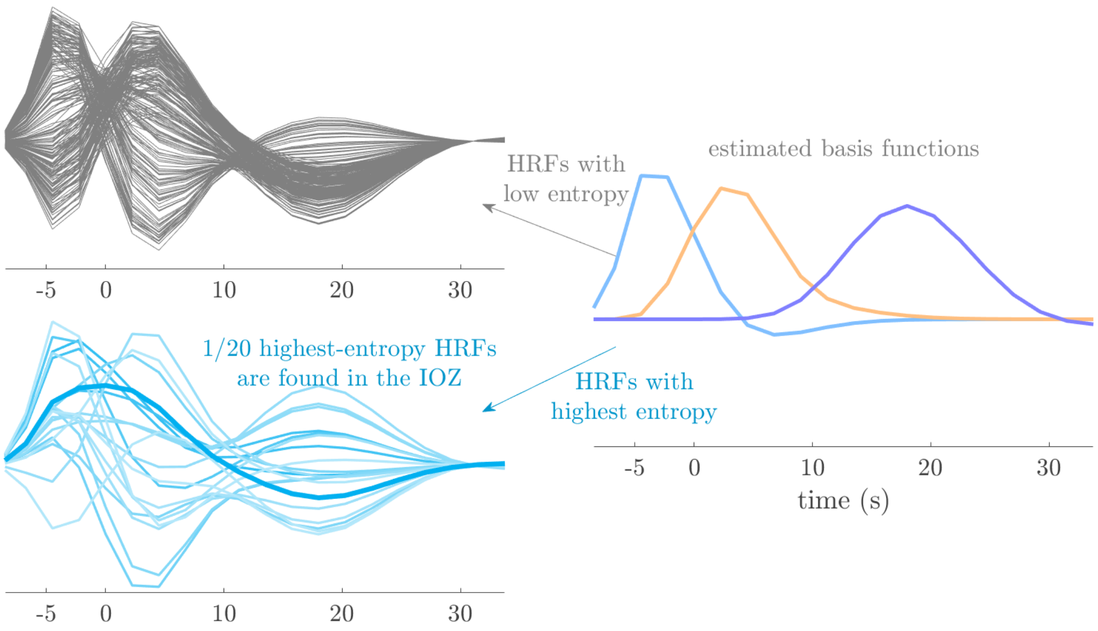

(b) Estimated HRFs with low and high entropy

Figure 11 Patient 7's estimated sources and neurovascular coupling parameters. **(b)** One of the ROIs with the highest-entropy HRFs belongs to the ictal onset zone (bold line, $p = 0.57$).

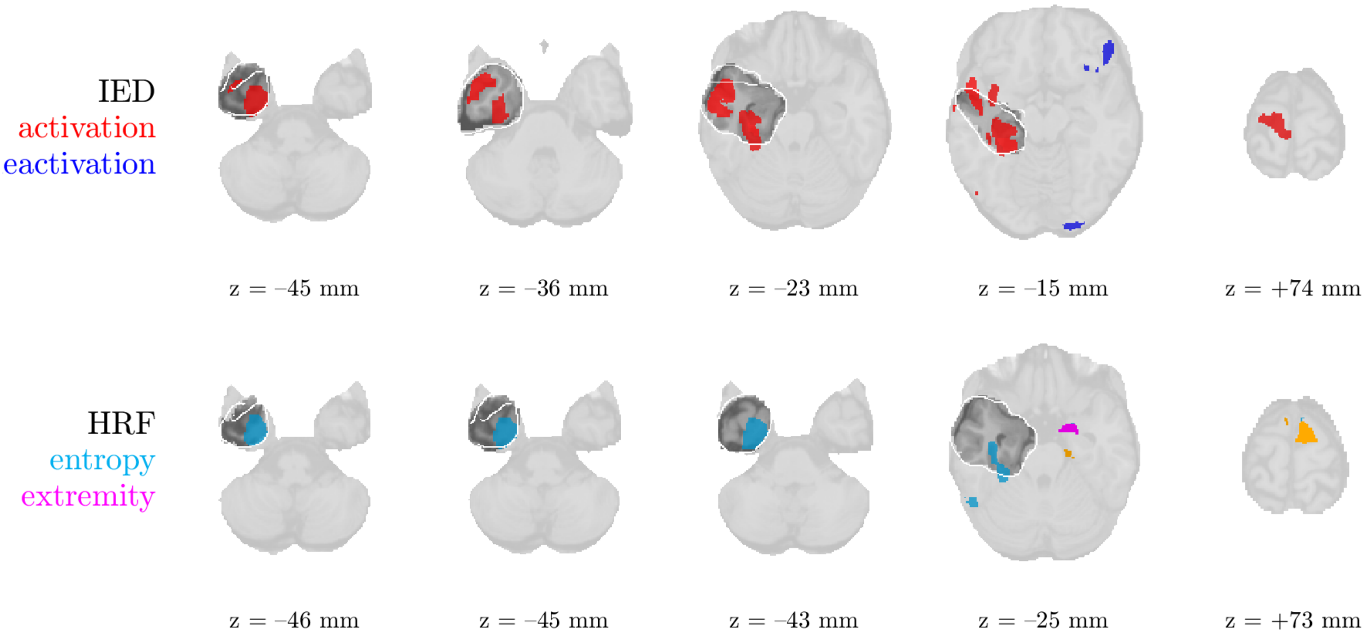

Figure 12 Patient 7's statistical nonparametric maps and HRF entropy/extremity maps. The ground truth ictal onset zone is highlighted in dark gray with a white contour. Regions with both high HRF entropy and high HRF extremity are colored in orange.

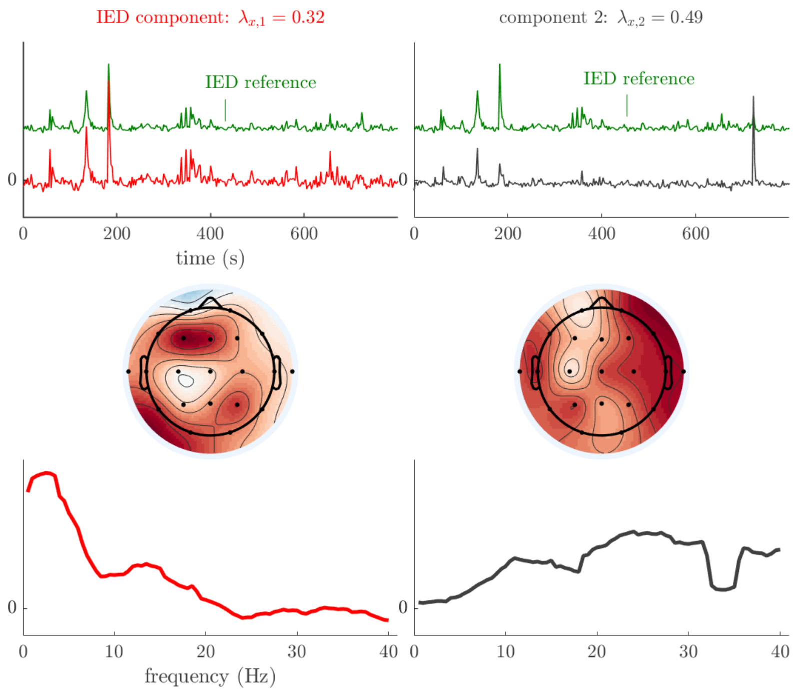

(a) Temporal ($\mathbf{s}_r$, top), spatial ($\mathbf{m}_r$, middle), and spectral ($\mathbf{g}_r$, bottom) profiles of the 2 sources in the EEG domain, and reference IED time course ($\mathbf{s}_{\text{ref}}$, in green).

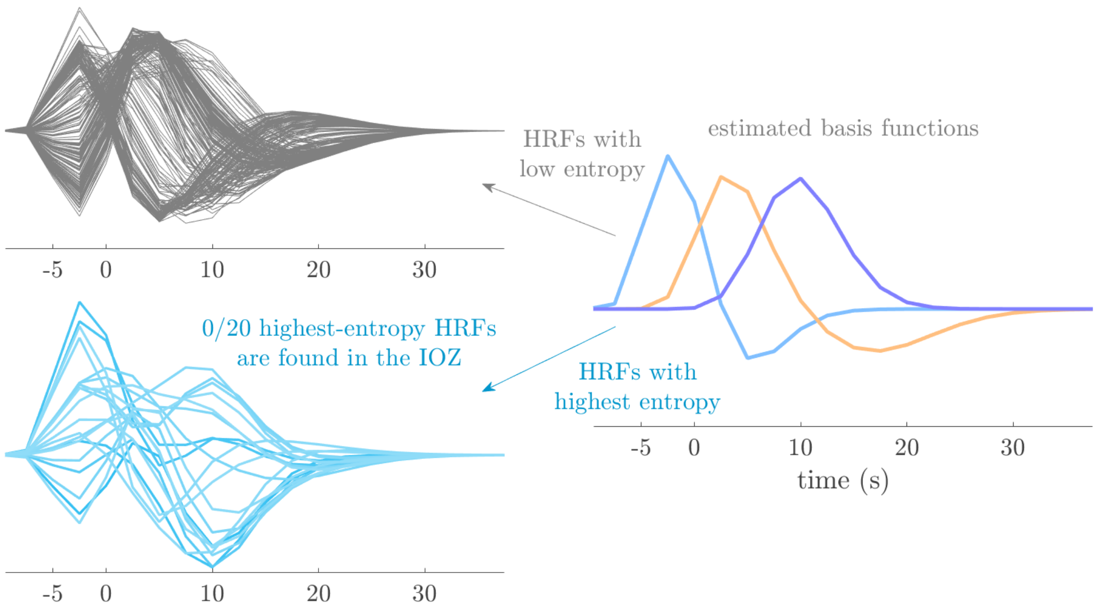

(b) Estimated HRFs with low and high entropy

Figure 13 Patient 8's estimated sources and neurovascular coupling parameters. **(b)** None of the ROIs with the highest-entropy HRFs belong to the ictal onset zone.

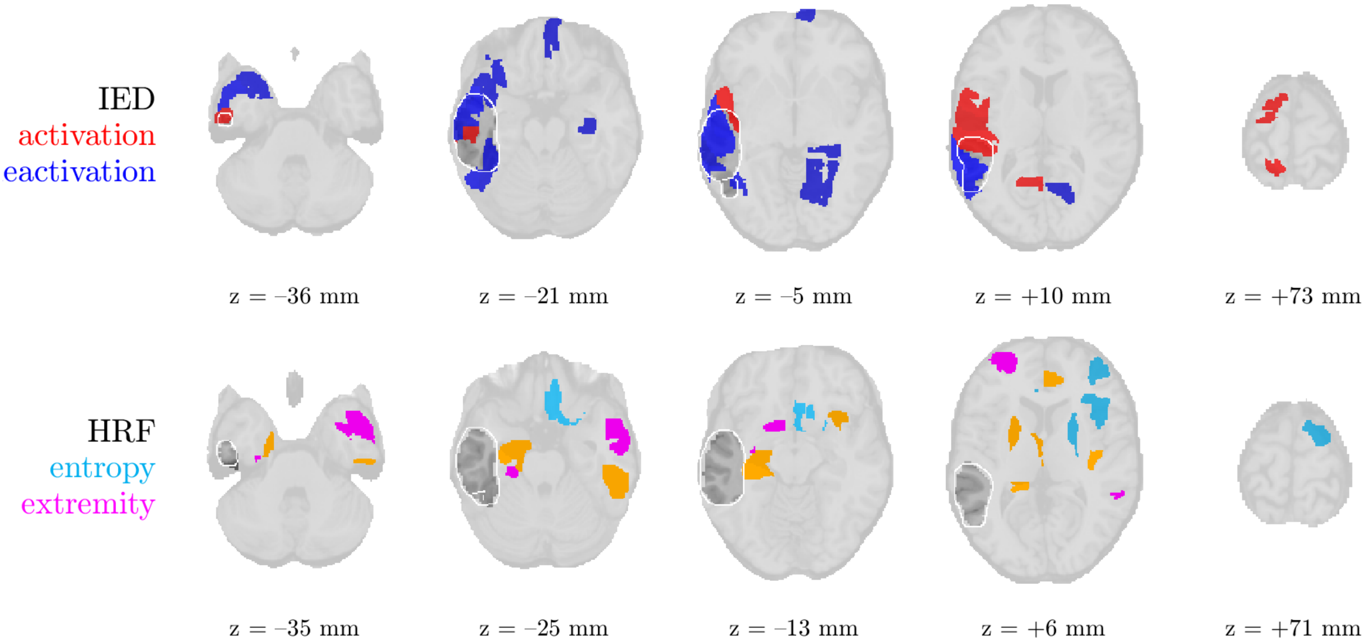

Figure 14 Patient 8's statistical nonparametric maps and HRF entropy/extremity maps. The ground truth ictal onset zone is highlighted in dark gray with a white contour. Regions with both high HRF entropy and high HRF extremity are colored in orange.

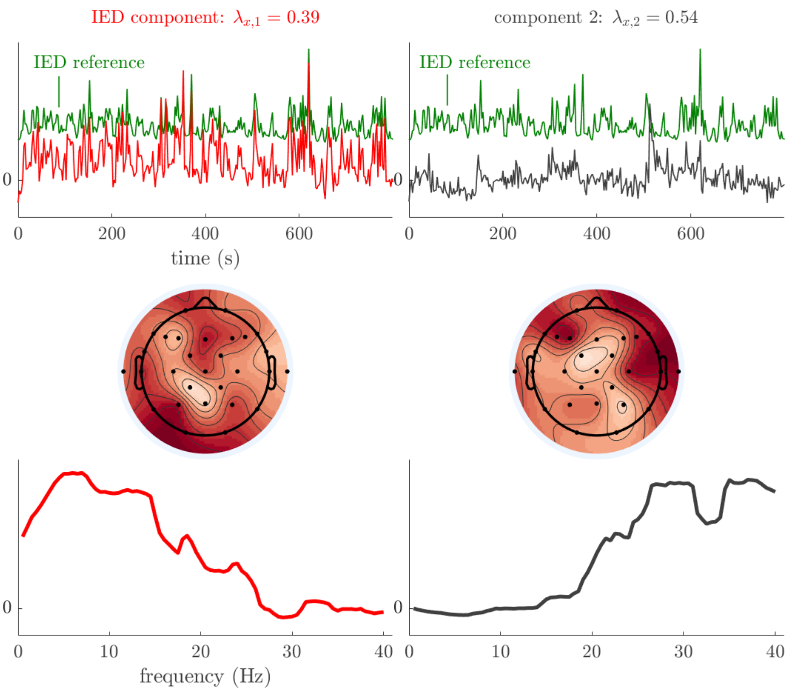

(a) Temporal ($\mathbf{s}_r$, top), spatial ($\mathbf{m}_r$, middle), and spectral ($\mathbf{g}_r$, bottom) profiles of the 2 sources in the EEG domain, and reference IED time course ($\mathbf{s}_{\text{ref}}$, in green).

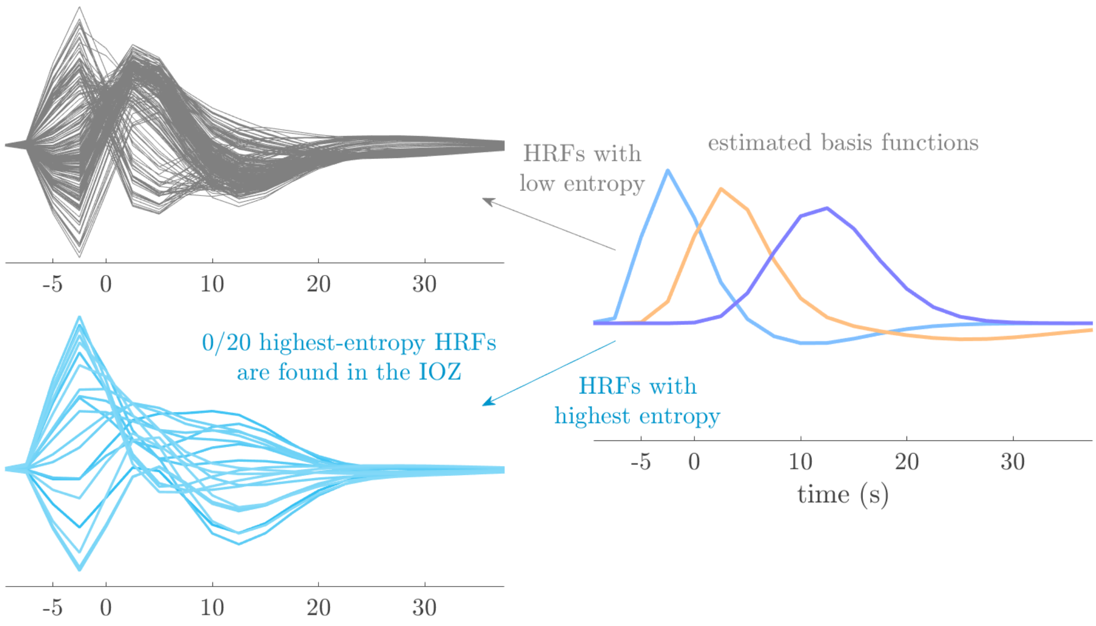

(b) Estimated HRFs with low and high entropy

Figure 15 Patient 9's estimated sources and neurovascular coupling parameters. **(b)** None of the ROIs with the highest-entropy HRFs belong to the ictal onset zone.

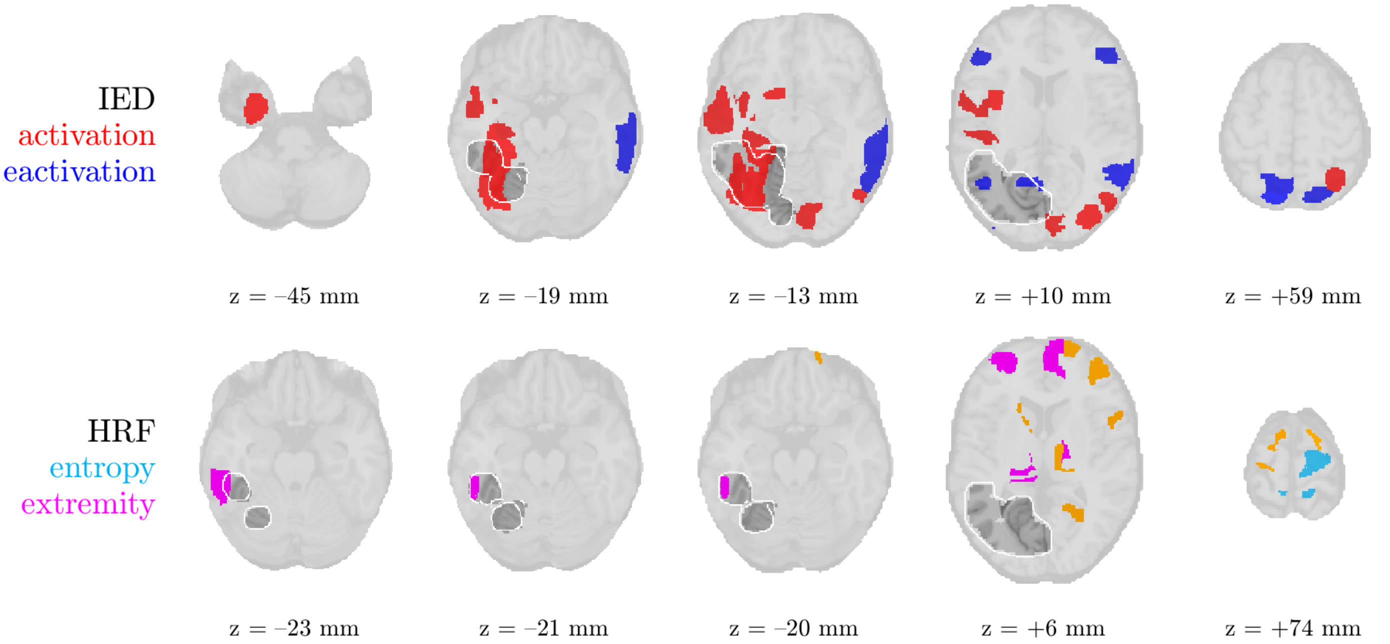

Figure 16 Patient 9's statistical nonparametric maps and HRF entropy/extremity maps. The ground truth ictal onset zone is highlighted in dark gray with a white contour. Regions with both high HRF entropy and high HRF extremity are colored in orange.

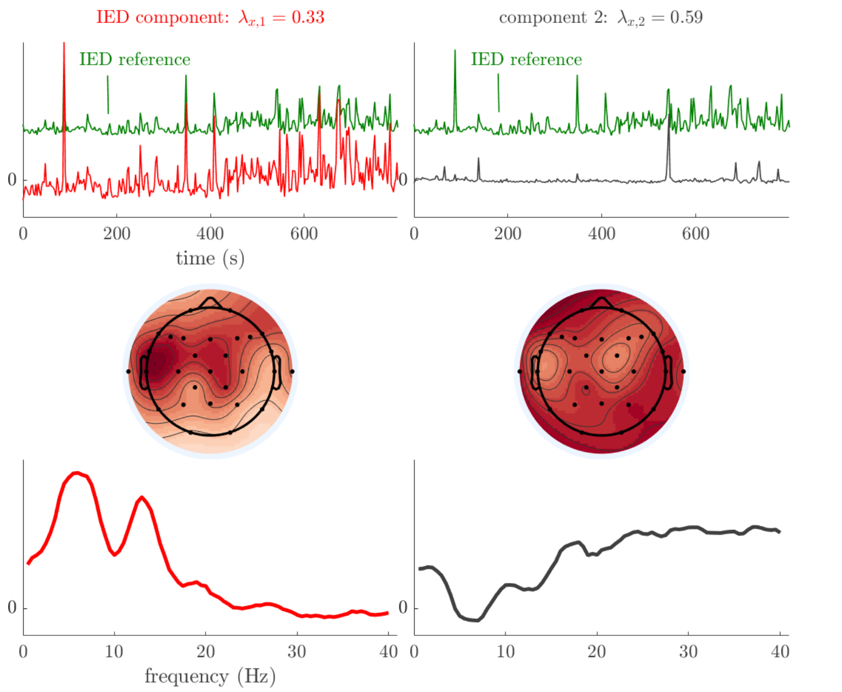

(a) Temporal ($\mathbf{s}_r$, top), spatial ($\mathbf{m}_r$, middle), and spectral ($\mathbf{g}_r$, bottom) profiles of the 2 sources in the EEG domain, and reference IED time course ($\mathbf{s}_{\text{ref}}$, in green).

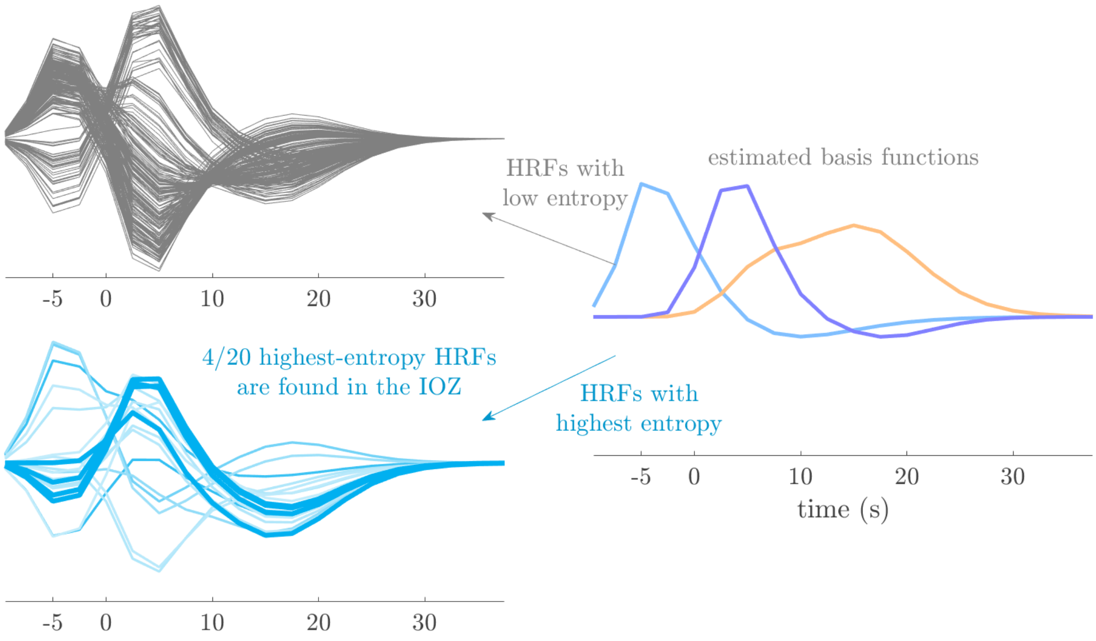

(b) Estimated HRFs with low and high entropy

Figure 17 Patient 11's estimated sources and neurovascular coupling parameters. **(b)** Four of the ROIs with the highest-entropy HRFs belong to the ictal onset zone (bold line, $p = 0.01$).

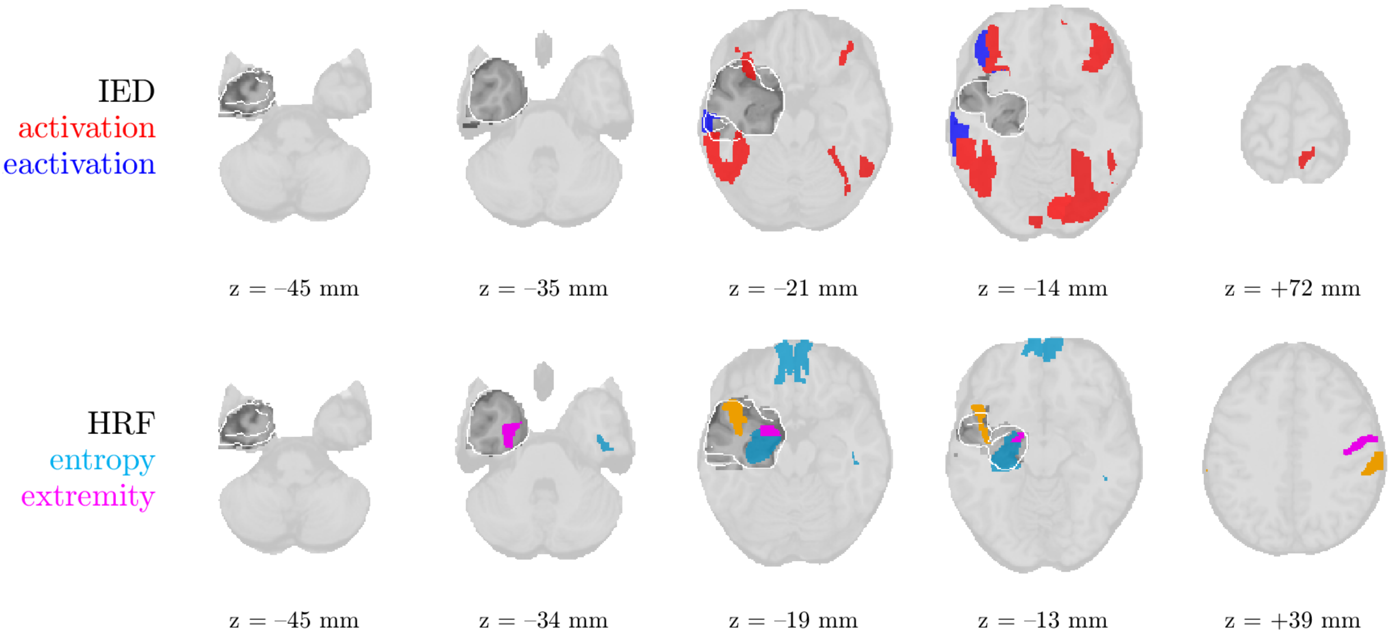

Figure 18 Patient 11's statistical nonparametric maps and HRF entropy/extremity maps. The ground truth ictal onset zone is highlighted in dark gray with a white contour. Regions with both high HRF entropy and high HRF extremity are colored in orange.

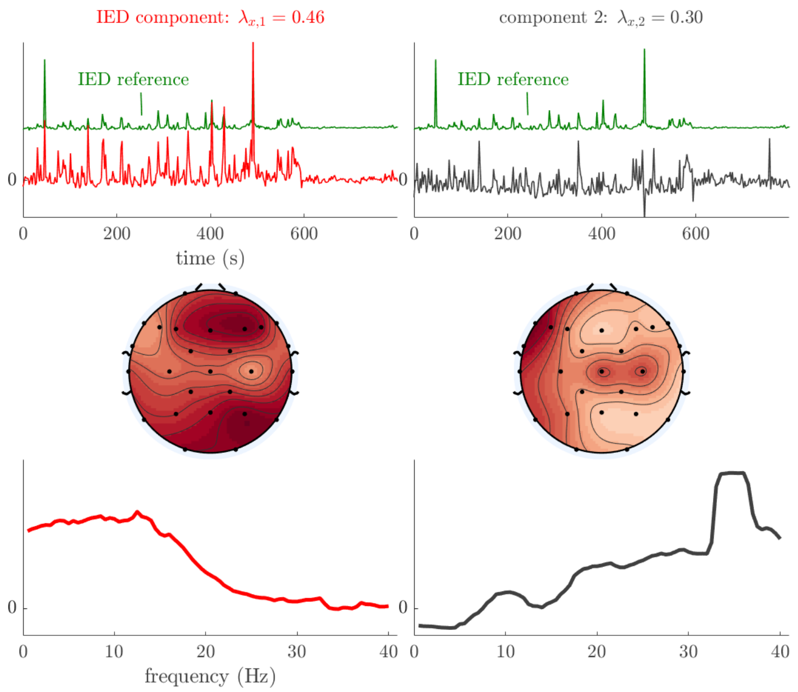

(a) Temporal ($\mathbf{s}_r$, top), spatial ($\mathbf{m}_r$, middle), and spectral ($\mathbf{g}_r$, bottom) profiles of the 3 sources in the EEG domain, and reference IED time course ($\mathbf{s}_{\text{ref}}$, in green).

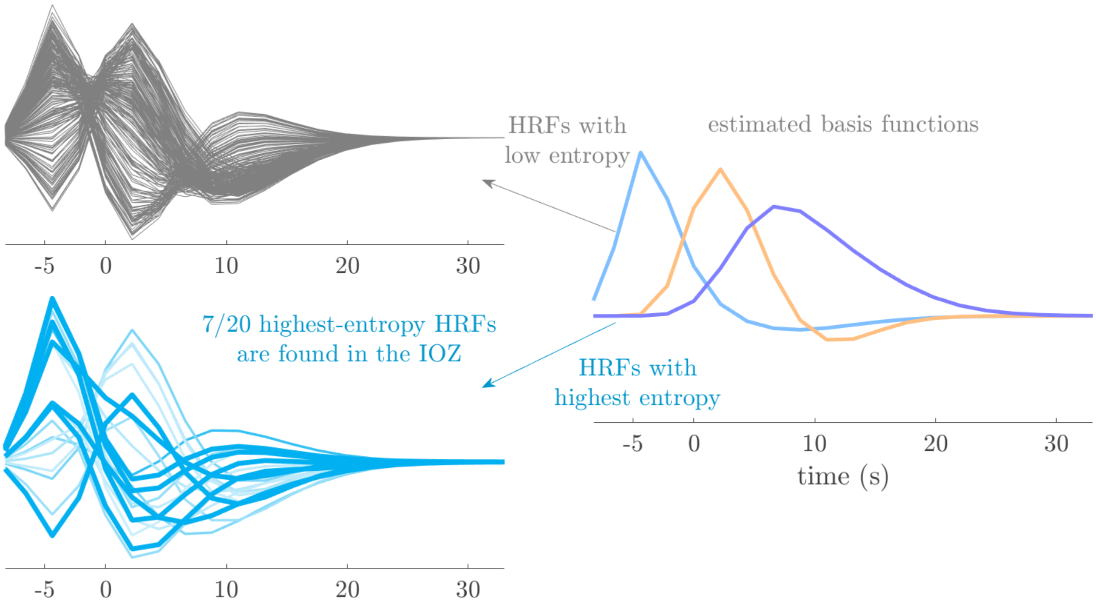

(b) Estimated HRFs with low and high entropy

Figure 19 Patient 12's estimated sources and neurovascular coupling parameters. **(b)** Seven of the ROIs with the highest-entropy HRFs belong to the ictal onset zone (bold line, $p < 10^{-3}$).

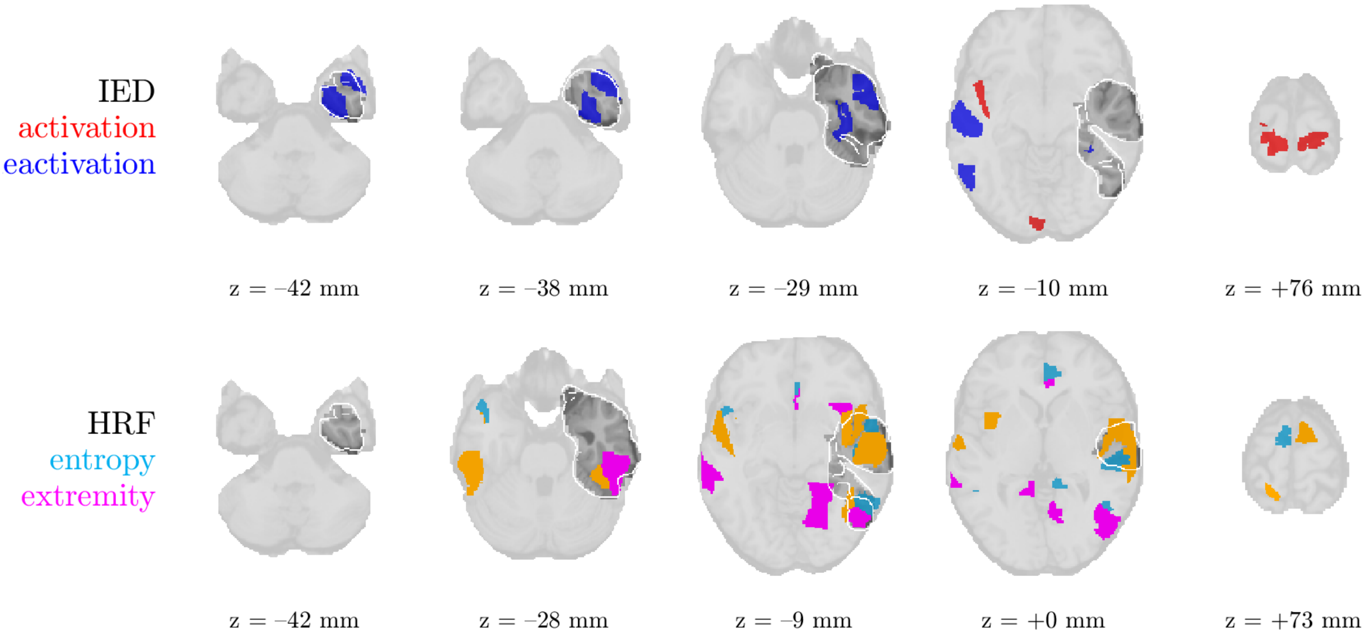

Figure 20 Patient 12's statistical nonparametric maps and HRF entropy/extremity maps. The ground truth ictal onset zone is highlighted in dark gray with a white contour. Regions with both high HRF entropy and high HRF extremity are colored in orange.
