# Supplementary material for: Augmenting interictal mapping with neurovascular coupling biomarkers by structured factorization of epileptic EEG and fMRI data
Source: Neuroimage. 2021 Mar;228:117652. doi: 10.1016/j.neuroimage.2020.117652 (PMC7903163; doi:10.1016/j.neuroimage.2020.117652)
Supplement: Supplementary Data S2 — Supplementary Raw Research Data. This is open data under the CC BY license http://creativecommons.org/licenses/by/4.0/ [file mmc2.pdf]

Augmenting interictal mapping with neurovascular coupling biomarkers by structured factorization of epileptic EEG and fMRI data

Simon Van Eyndhoven[a,*], Patrick Dupont[b,c], Simon Tousseyn[d], Nico Vervliet[a], Wim Van Paesschen[e,f], Sabine Van Huffel[a], Borbála Hunyadi[g]

[a]KU Leuven, Department of Electrical Engineering (ESAT), STADIUS Center for Dynamical Systems, Signal Processing and Data Analytics
[b]Laboratory for Cognitive Neurology, Department of Neurosciences, KU Leuven, Leuven, Belgium
[c]Leuven Brain Institute, Leuven, Belgium
[d]Academic Center for Epileptology, Kempenhaeghe and Maastricht UMC+, Heeze, The Netherlands
[e]Laboratory for Epilepsy Research, KU Leuven, Leuven, Belgium
[f]Department of Neurology, University Hospitals Leuven, Leuven, Belgium
[g]Circuits and Systems Group (CAS), Department of Microelectronics, Delft University of Technology, Delft, the Netherlands

Supplement 2: Effect of order of preprocessing steps

Based on valuable input from the reviewers, we analyzed post-hoc whether there was an adverse effect of carrying out some fMRI preprocessing steps sequentially, instead of simultaneously. Specifically, there was concern that the effect of nuisance regression could have been partially undone by the later band-pass filtering step.

In our pipeline, all nuisance regressors (including dummy regressors for the scans with large head motion, cfr. Section 2.2 in the main text) were collected into one large set, which was simultaneously regressed out from the BOLD signals. Afterwards, the BOLD signals underwent band-pass filtering, which was performed in the frequency domain using the CONN toolbox. Based on inspection of the power spectra of the final BOLD signals that served as input to the factorization, we confirmed that the band-pass filtering was successful.

Post-hoc, we investigated the impact on the removal of nuisance regressors (covariates of no intererst) by the steps that came afterwards (i.e., band-pass filtering). For each nuisance regressor, we analyzed its correlation with the BOLD signals at the following 4 steps:

1. At the start of the pipeline
2. right after regressing it out using orthogonal projection (this serves as a sanity check, since these correlations should by construction be zero)
3. after band-pass filtering
4. after averaging within each ROI of the atlas

We summarize the correlations for each patient in Figure 1. As expected, in the beginning there are very large correlations with some nuisance regressors, which are fully neutralized after the regression step. At the end of the pipeline, after band-pass filtering and atlas-based averaging, the correlations are very close to, but not exactly equal to zero.

Hence, this analysis confirms that caution for a (partial) reintroduction of correlations with nuisance regressions is warranted. However, the spurious correlations which exist at the end of the pipeline are very minor, hence we believe that our results are not badly compromised by this unwanted effect.

*Corresponding author
Email address: simon.vaneyndhoven@kuleuven.be, simon.vaneyndhoven@gmail.com

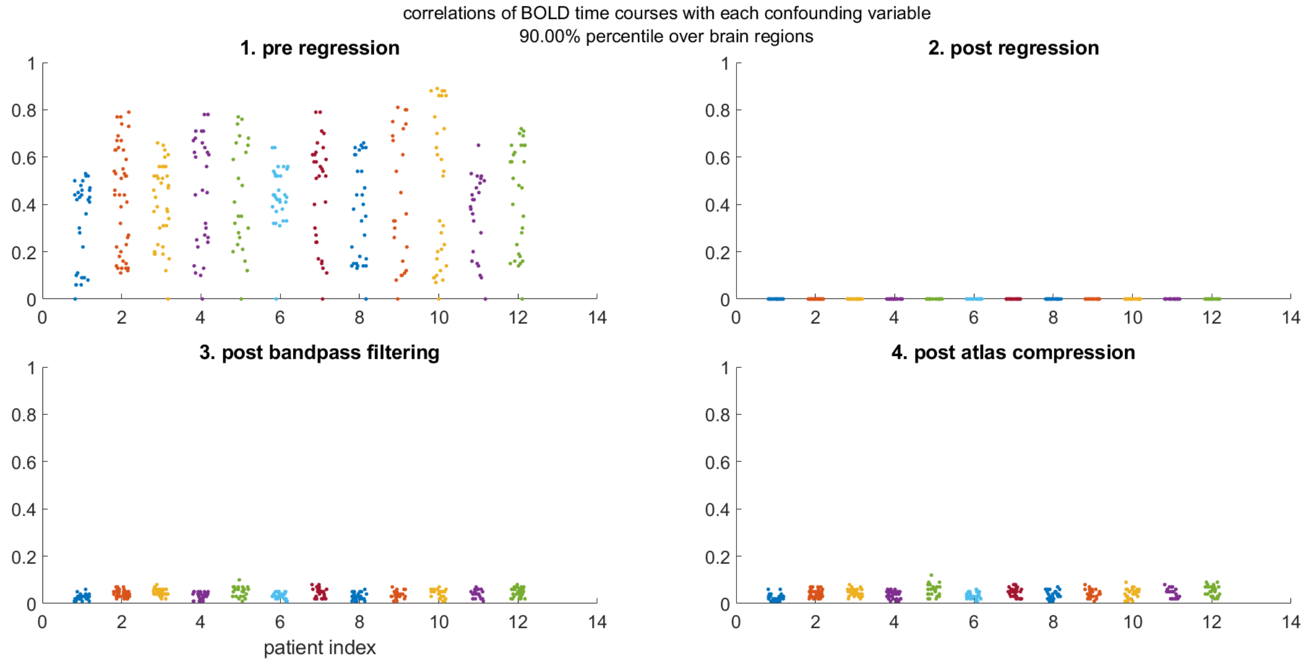

Figure 1 Performing nuisance regression and filtering sequentially, instead of simultaneously, can partially undo the effect of nuisance regression, since spurious correlations with covariates of no interest can be reintroduced by the band-pass filtering step.
