# Supplementary material for: Augmenting interictal mapping with neurovascular coupling biomarkers by structured factorization of epileptic EEG and fMRI data
Source: Neuroimage. 2021 Mar;228:117652. doi: 10.1016/j.neuroimage.2020.117652 (PMC7903163; doi:10.1016/j.neuroimage.2020.117652)
Supplement: Supplementary Data S3 — Supplementary Raw Research Data. This is open data under the CC BY license http://creativecommons.org/licenses/by/4.0/ [file mmc3.pdf]

# Augmenting interictal mapping with neurovascular coupling biomarkers by structured factorization of epileptic EEG and fMRI data

Simon Van Eyndhoven[a,*], Patrick Dupont[b,c], Simon Tousseyn[d], Nico Vervliet[a], Wim Van Paesschen[e,f], Sabine Van Huffel[a], Borbála Hunyadi[g]

[a]*KU Leuven, Department of Electrical Engineering (ESAT), STADIUS Center for Dynamical Systems, Signal Processing and Data Analytics*
[b]*Laboratory for Cognitive Neurology, Department of Neurosciences, KU Leuven, Leuven, Belgium*
[c]*Leuven Brain Institute, Leuven, Belgium*
[d]*Academic Center for Epileptology, Kempenhaeghe and Maastricht UMC+, Heeze, The Netherlands*
[e]*Laboratory for Epilepsy Research, KU Leuven, Leuven, Belgium*
[f]*Department of Neurology, University Hospitals Leuven, Leuven, Belgium*
[g]*Circuits and Systems Group (CAS), Department of Microelectronics, Delft University of Technology, Delft, the Netherlands*

## Supplement 3: Statistical maps from EEG-correlated fMRI analysis

As a benchmark for the results in the current paper, we show parametric statistical activation maps obtained via a classical EEG-correlated fMRI analysis via the general linear model (GLM), which we conducted for the same patient cohort in (Van Eyndhoven et al., 2019). We computed t-maps for significant activation of interictal epileptic discharges (IEDs), for which we constructed an IED predictor time course for each patient. This predictor coincides with the reference time course in Appendix B.3, which we use for selection of the IED-related component in the coupled matrix-tensor factorization in the present study. It is computed by processing the subject's EEG signals with a multi-channel Wiener filter (cfr. Section 2.3), subsequently squaring the output, and computing the average time course over all channels. In our previous study, we found that such a representation of the IEDs led to the most sensitive results for detecting the interictal onset zone, while maintaining maximal specificity (Van Eyndhoven et al., 2019).

For each patient, we show the significant activation in the same axial slices that are used in the main manuscript and Supplement 1. The highlighted regions are thresholded at z-score of 3.4, and meet a cluster size lower bound of 2800 $mm^3$ (Tousseyn et al., 2014).

## References

Tousseyn, S., Dupont, P., Goffin, K., Sunaert, S., & Van Paesschen, W. (2014). Sensitivity and specificity of interictal EEG-fMRI for detecting the ictal onset zone at different statistical thresholds. *Frontiers in neurology*, *5*, 131.

Van Eyndhoven, S., Hunyadi, B., Dupont, P., Van Paesschen, W., & Van Huffel, S. (2019). Semi-automated EEG enhancement improves localization of ictal onset zone with EEG-correlated fMRI. *Frontiers in neurology*, *10*.

*Corresponding author
*Email address:* simon.vaneyndhoven@kuleuven.be, simon.vaneyndhoven@gmail.com

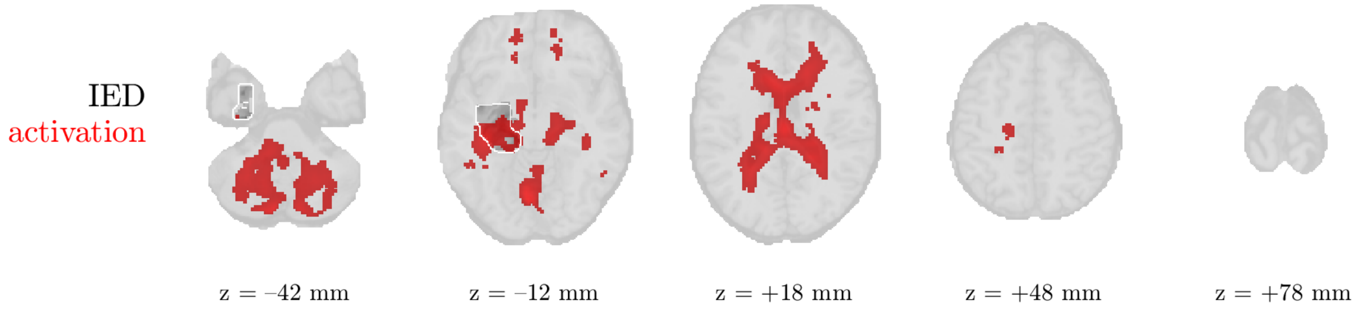


**Figure 1** Patient 1's statistical parametric activation map, obtained via the standard GLM for IED activation. The ground truth ictal onset zone is highlighted in dark gray with a white contour.

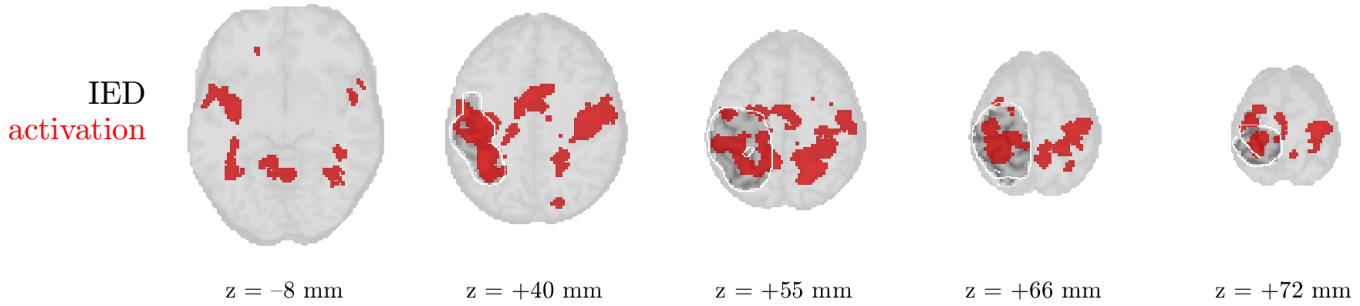


**Figure 2** Patient 2's statistical parametric activation map, obtained via the standard GLM for IED activation. The ground truth ictal onset zone is highlighted in dark gray with a white contour.

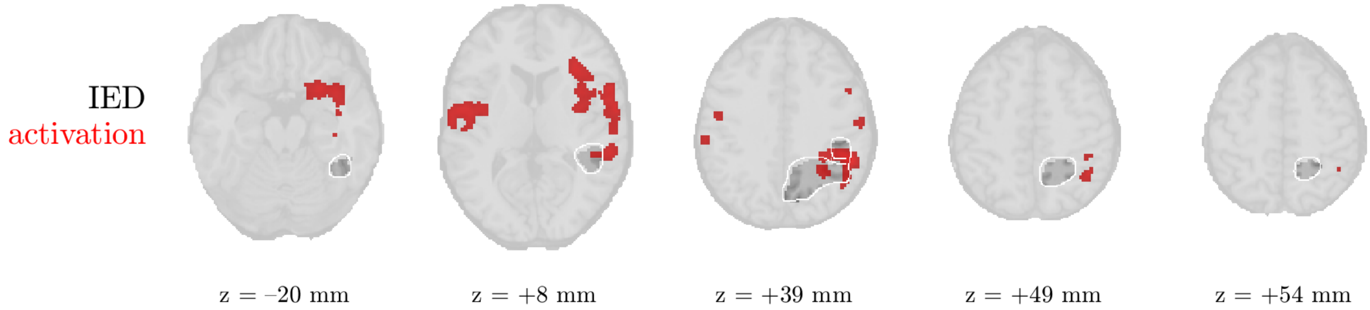


**Figure 3** Patient 3's statistical parametric activation map, obtained via the standard GLM for IED activation. The ground truth ictal onset zone is highlighted in dark gray with a white contour.

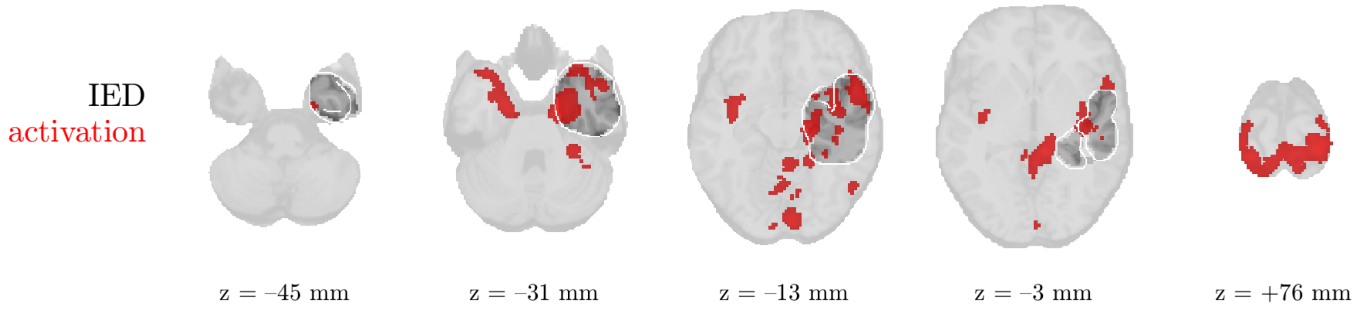


**Figure 4** Patient 4's statistical parametric activation map, obtained via the standard GLM for IED activation. The ground truth ictal onset zone is highlighted in dark gray with a white contour.

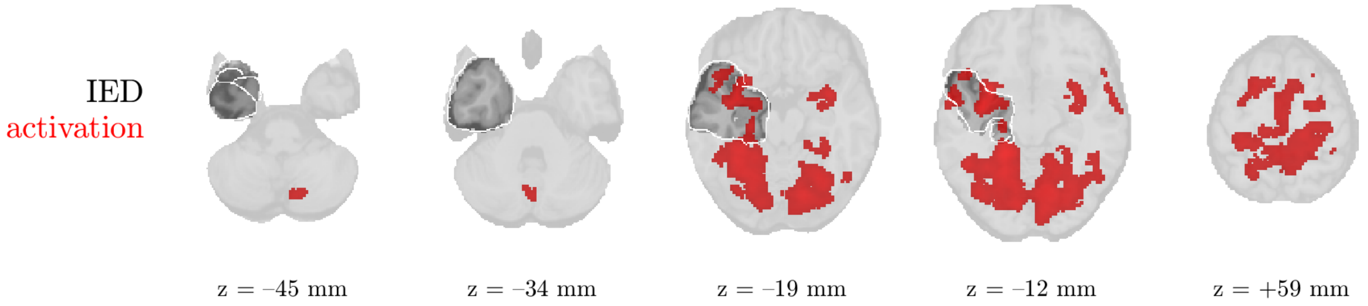


**Figure 5** Patient 5's statistical parametric activation map, obtained via the standard GLM for IED activation. The ground truth ictal onset zone is highlighted in dark gray with a white contour.

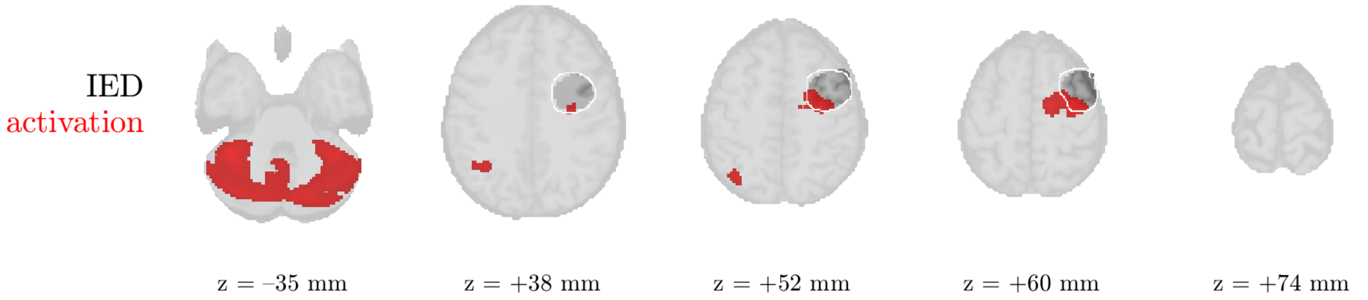


**Figure 6** Patient 6's statistical parametric activation map, obtained via the standard GLM for IED activation. The ground truth ictal onset zone is highlighted in dark gray with a white contour.

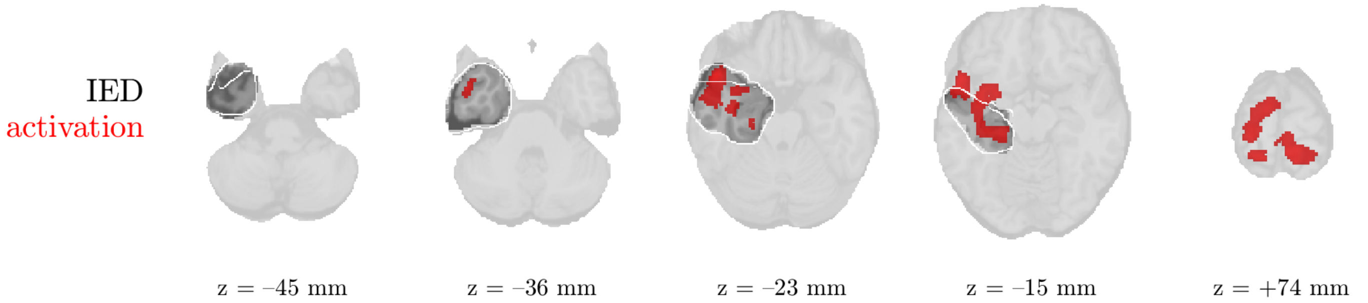


**Figure 7** Patient 7's statistical parametric activation map, obtained via the standard GLM for IED activation. The ground truth ictal onset zone is highlighted in dark gray with a white contour.

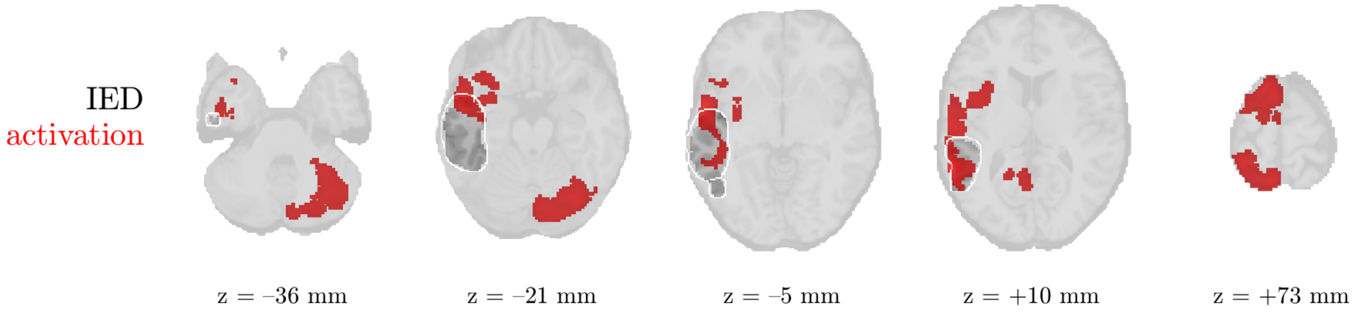


**Figure 8** Patient 8's statistical parametric activation map, obtained via the standard GLM for IED activation. The ground truth ictal onset zone is highlighted in dark gray with a white contour.

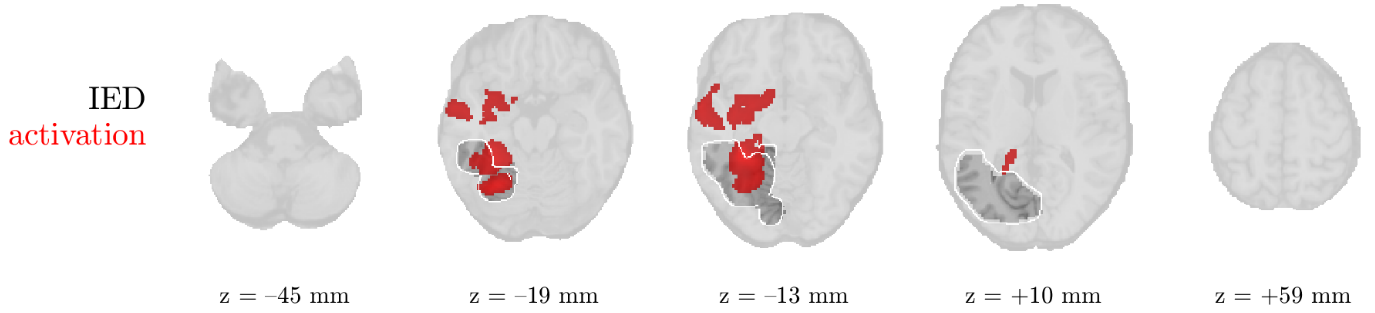


**Figure 9** Patient 9's statistical parametric activation map, obtained via the standard GLM for IED activation. The ground truth ictal onset zone is highlighted in dark gray with a white contour.

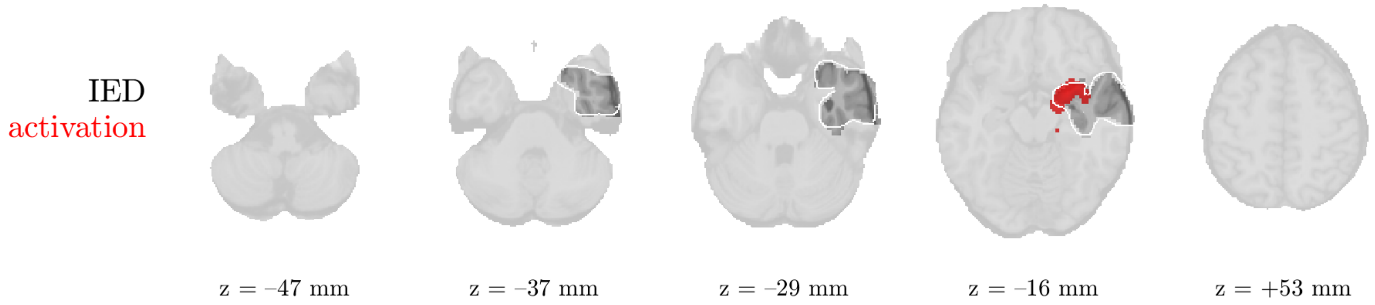


**Figure 10** Patient 10's statistical parametric activation map, obtained via the standard GLM for IED activation. The ground truth ictal onset zone is highlighted in dark gray with a white contour.

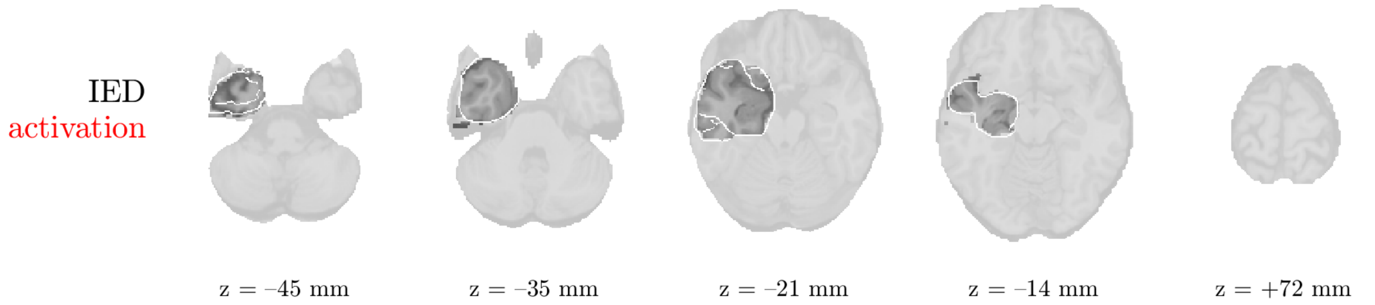


**Figure 11** Patient 11's statistical parametric activation map, obtained via the standard GLM for IED activation. The ground truth ictal onset zone is highlighted in dark gray with a white contour.

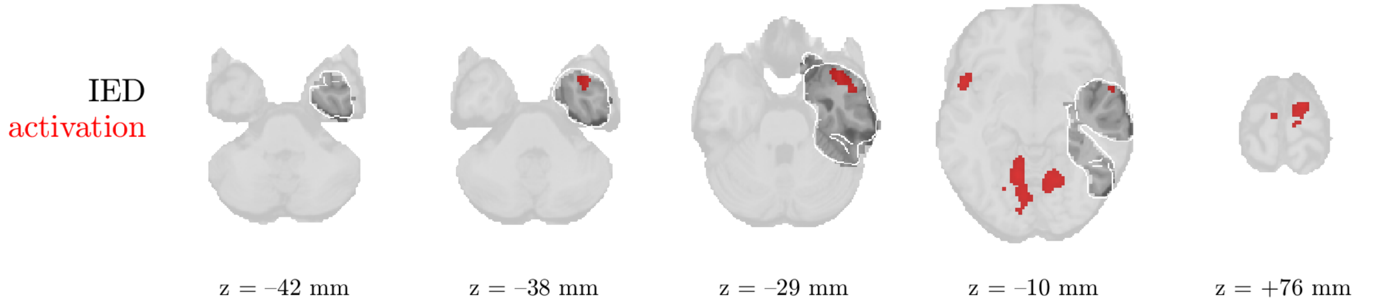


**Figure 12** Patient 12's statistical parametric activation map, obtained via the standard GLM for IED activation. The ground truth ictal onset zone is highlighted in dark gray with a white contour.
